# Differential Gene Expression with an Emphasis on Floral Organ Size Differences in Natural and Synthetic Polyploids of *Nicotiana tabacum* (Solanaceae)

**DOI:** 10.3390/genes11091097

**Published:** 2020-09-19

**Authors:** Jacob B. Landis, Amelda Kurti, Amber J. Lawhorn, Amy Litt, Elizabeth W. McCarthy

**Affiliations:** 1 Department of Botany and Plant Sciences, University of California Riverside, Riverside, CA 92521, USA; ameldakurti@gmail.com (A.K.); alawh002@ucr.edu (A.J.L.); amy.litt@ucr.edu (A.L.); 2 School of Integrative Plant Science, Section of Plant Biology and the L.H. Bailey Hortorium, Cornell University, Ithaca, NY 14853, USA; 3 Department of Biology, SUNY Cortland, Cortland, NY 13045, USA

**Keywords:** Nanopore, GASA, BIG BROTHER, flower size, cell size, RNA-Seq

## Abstract

Floral organ size, especially the size of the corolla, plays an important role in plant reproduction by facilitating pollination efficiency. Previous studies have outlined a hypothesized organ size pathway. However, the expression and function of many of the genes in the pathway have only been investigated in model diploid species; therefore, it is unknown how these genes interact in polyploid species. Although correlations between ploidy and cell size have been shown in many systems, it is unclear whether there is a difference in cell size between naturally occurring and synthetic polyploids. To address these questions comparing floral organ size and cell size across ploidy, we use natural and synthetic polyploids of *Nicotiana tabacum* (Solanaceae) as well as their known diploid progenitors. We employ a comparative transcriptomics approach to perform analyses of differential gene expression, focusing on candidate genes that may be involved in floral organ size, both across developmental stages and across accessions. We see differential expression of several known floral organ candidate genes including *ARF2*, *BIG BROTHER*, and *GASA/GAST1*. Results from linear models show that ploidy, cell width, and cell number positively influence corolla tube circumference; however, the effect of cell width varies by ploidy, and diploids have a significantly steeper slope than both natural and synthetic polyploids. These results demonstrate that polyploids have wider cells and that polyploidy significantly increases corolla tube circumference.

## 1. Introduction

Organ growth is highly regulated, controlled by both intrinsic and extrinsic factors [1]. Organ size is controlled by either cell growth or cell proliferation, with the combination of the two often being the highest contributor [2]. Cell proliferation occurs early in development followed by cell expansion [3,4], with an indication that cell division and organ growth are to a certain degree independent processes [5]. However, a few studies have shown a co-regulation of cell division and expansion, albeit with different genetic pathways [6,7,8]. The relative contribution of each phase is variable among species [9,10], likely contributing to different mating systems and pollinator syndromes [11,12,13,14]. Flower size has important ecological implications such as pollinator visitation and pollination success [15,16,17] in wild and domesticated plants (as reviewed in [18]). Across angiosperms, closely related species have similar floral architecture in general, yet the size and shape can be highly labile and highly heritable [19].

The genetic components governing cell number, size, and shape in floral organs are not well understood [20,21] though a hypothesized pathway has been described previously [2,18,22]. Candidate genes have been identified in model organisms (i.e., *Arabidopsis thaliana*, *Antirrhinum majus*, and *Petunia hybrida*) associated with organ size including GA-stimulated transcript *GASA/GAST1/GEG* [23,24,25], *BIG BROTHER* (*BB*; [26]), *BIG PETAL* (*BPE*; [27]), *LONGIFOLIA* (*LNG*; [28]), *ANGUSTIFOLIA* (*AN*; [29]), *ARGOS* [30,31], *AINTEGUMENTA* (*ANT*; [32,33]), *AUXIN RESPONSE FACTOR2 (ARF2*; [34]), *KLUH* [35], *EBP1* [36], *DA1/DAR1* [37,38], *RAMOSA1* (*Ra1*; [39]), *BIGGER ORGANS* (*BIO*; [40]), and *ELEPHANT-EAR-LIKE LEAF1* (*ELE1*; [40]). The majority of candidate genes have been shown to be promoters or activators of elongation (*GASA/GAST1/GEG*, *LNG*, *AN*, *ARGOS*, *ANT*, *KLUH*, and *Ra1*) or promoters of cell proliferation (*ARF2*). Others have been shown to be repressors or inhibitors of growth (*BB*, *BPE*, *BIO*, and *ELE1*) or cell division (*DA1/DAR1*). Even though the prevalence of floral based transcriptomic studies has increased in recent years [41,42,43,44,45], only a limited number of studies have focused on genes associated with flower size differences in non-model taxa including studies in *Annona* [46], *Chrysanthemum* [47], *Lithospermum* [48], *Populus* [49], and *Saltugilia* [50]. 

Polyploidy, or whole genome duplication, is prevalent across the history of land plants [51] and is associated with increased diversification in the angiosperms [52] and in smaller clades of ferns [53]. Following polyploidy events, functionally important genes, such as those allowing for rapid adaptation to environmental change, are often preferentially retained [54], promoting species diversification through ecological niche shifts [55,56]. There is a strong positive correlation between genome size and cell size [57,58,59,60,61], yet the relationship is not consistent in all cell types [62]. Increases in the cell size of polyploids compared to diploids has been observed [63,64,65], and endoreduplication within a single individual often yields larger cells [66,67,68,69,70]. Genome size has been shown to be a crucial component in determining the minimum size of the cell and represents the upper limit for cell packing densities, which is crucial for carbon assimilation and important in resource allocation for growth, reproduction, and defense [61,71].

While natural polyploids have been used to investigate evolutionary and ecological questions including ecological niche differentiation, defense against herbivores, and stress tolerance [72,73,74,75,76,77], the production of synthetic allopolyploids allows for the study of traits instantaneously after genome merger. Many recent papers have used synthetic polyploids to study functional traits and inflorescence morphology in a variety of species [78,79,80,81,82,83]. By comparing natural and synthetic polyploids, the immediate consequences of polyploidy can be disentangled from the changes that occur via subsequent evolution following whole genome duplication.

*Nicotiana* is an excellent system to study polyploidy because approximately half of the species are allotetraploids which arose via six polyploidy events [84,85,86,87,88,89,90]. Polyploids of different ages, along with available synthetic lines created from the same progenitor species as natural polyploids, allow for investigation into the consequences of immediate, short-term, and long-term polyploid evolution. In addition, *Nicotiana* displays wide variation in corolla tube length and width [91]. Young *Nicotiana* polyploids (<1 million years old (myo)) tend to evolve shorter and wider corolla tubes than what would be expected based on the intermediate values of their diploid progenitors, whereas diploids and older polyploids (> 1 myo) do not show any trends in corolla tube size evolution [90].

Within the broader Solanaceae, the breeding system appears to have the largest impact on diversification patterns [92]. However, increased speciation is associated with older polyploid events compared to younger polyploid clades in *Nicotiana* [87], and diversification in *Petunia* (Solanaceae) is largely driven by flower size, with flower size directly influencing pollinator system [93,94,95]. The disconnect between diversification patterns at the family level and at the genus level provides additional support for studying comparative biology in model clades to gain a deeper understanding of underlying mechanisms [96,97].

Most studies investigating the genetics of flower size have focused on model organisms. There is a large gap in our understanding across the angiosperm tree of life in terms of which genes are responsible for observed differences in floral size and if any clade specific patterns exist. Here, we use *Nicotiana* polyploids and their progenitors to address the following questions related to changes in floral organ size. Are increases in tube width of polyploids due to increased cell width compared to their diploid progenitors? Do the natural and synthetic polyploids of *N. tabacum* show similar patterns of differential gene expression, both within and among taxa at specific developmental time points? Do any of the known floral size candidate genes show differential expression? Do we see clear associations between organ size genes, overall flower size, and changes in cell size? The analyses presented here will advance our understanding of floral evolution after whole genome duplication and the association of genome size and cell size in floral tissue while also providing an additional comparison to determine if known candidate genes impacting floral organ size show similar patterns of differential expression to previously investigated taxa.

## 2. Material and Methods

### 2.1. Plant Material

**Figure 1:**
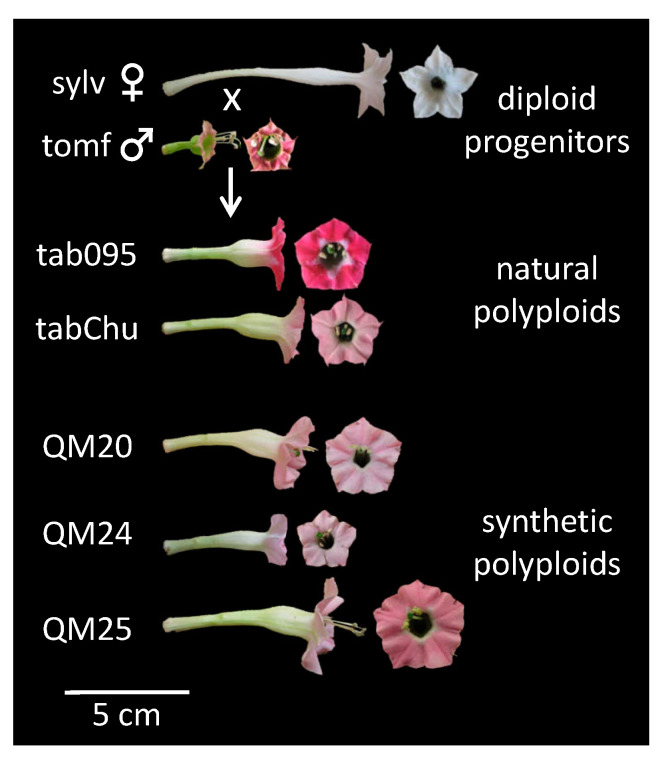
Floral morphology of diploid progenitors *N. sylvestris* and *N. tomentosiformis*, two natural *N. tabacum* accessions (095-55 and ‘Chulumani’), and three synthetic *N. tabacum* lines (QM20, QM24, and QM25): all accessions pictured here are included in the cell size data set, whereas all accessions except QM20 were used in the transcriptome analyses. Sylv = *N. sylvestris*, tomf = *N. tomentosiformis*, tab095 = *N. tabacum* 095-55, tabChu = *N. tabacum* ‘Chulumani’, QM20 = synthetic *N. tabacum* QM20, QM24 = synthetic *N. tabacum* QM24, and QM25 = synthetic *N. tabacum* QM25.

Plant material was grown in greenhouses with natural sunlight and temperatures between 10 and 30 °C at the University of California, Riverside. The following accessions were used: *Nicotiana sylvestris* A04750326 (Radboud University, Nijmegen, The Netherlands); *N. tomentosiformis* BRNO 4103 (acquired from A. Kovařík, Brno, Czech Republic); *N. tabacum* 095-55 (IPK Gatersleben, Germany); *N. tabacum* ‘Chulumani’ (collected in the field in Bolivia by S. Knapp); and three first-generation synthetic lines, QM20, QM24, and QM25 (created by K.Y. Lim at Queen Mary, University of London by crossing 4x autotetraploid *N. sylvestris* and 4x autotetraploid *N. tomentosiformis*; Figure 1). Because multiple accessions of *N. tabacum* were used throughout the study, we will refer to plant lines as accessions.

### 2.2. Flower Size and Cell Size

To measure differences in cell size and cell number, we stained fresh corolla tissue at anthesis in 0.1% aniline blue in 1N K_3_PO_4_ for 2 h. We collected the tissue from one half of the mouth of the corolla tube, just below the floral limb (Figure S1). Stained tissues were mounted on microscope slides and imaged on a Leica DM5500 B fluorescent microscope with the DAPI filter using tiling to create a mosaic image of the entire tissue. We measured corolla tube circumference using Fiji [98] with the length of the mounted tissue just below the floral limb (Figure S1) serving as a proxy for corolla tube width. Number of cells was also counted, and the width of 100 consecutive cells were measured, starting at the dorsal side of the flower. We used four flowers each from *N. sylvestris*; *N. tomentosiformis*; *N. tabacum* 095-55; *N. tabacum* ‘Chulumani’; and the synthetic *N. tabacum* lines QM20, QM24, and QM25.

To determine if differences in cell size, cell number, and ploidy influence the corolla tube circumference at the corolla mouth, we ran a Linear Model in R [99] using cell number, average cell width, and ploidy as predictor variables. To investigate which factors were significant in the model, we started by including two-way interactions and compared the fit of this model to that of models with a single factor removed using the drop1 function in the lmerTest package (version 3.0.1; [100]). We chose a reduced model (corolla tube circumference~width + ploidy + width:ploidy + cell number) in which all factors included had a significant effect on corolla tube circumference and only removed factors that did not alter the fit of the model. We checked the assumptions of normality and constant variance, and the data were appropriate. We used our chosen model to predict corolla tube circumference using the full range of cell width and cell number values in the dataset and calculated 95% confidence intervals (mean ± 2SE). We used the car::anova function to determine whether model variables significantly affected corolla tube circumference and also performed post hoc pairwise comparisons using the lsmeans function [101] to investigate the significant interaction between ploidy and cell width. In these pairwise analyses, we compared the corolla tube circumference of diploids, natural polyploids, and synthetic polyploids as predicted by our best model at minimum, 1st quartile, median, 3rd quartile, and maximum cell width values. We ran linear models to determine whether ploidy affects both cell width and cell number (width~ploidy and cell number~ploidy) and performed ANOVAs using the aov function with post hoc Tukey tests (α = 0.05) to determine whether diploids, natural polyploids, or synthetic polyploids had significantly different cell widths and cell numbers. We plotted the predicted data based on the continuous effects in R using the geom_smooth function in ggplot2 (version 3.0.0; [102]). We plotted the discrete effects as strip plots and plotted strip plots for cell width, cell number, and corolla tube circumference for all accessions. R scripts were uploaded to GitHub (https://github.com/elizabethwmccarthy/).

### 2.3. Transcriptome Sequencing and Analyses

Flower material from three developmental time points (60%, 85%, and 95% mean corolla length at anthesis including the floral limb, the part of the flower that opens, mean calculated from a minimum of five flowers per accession) from three biological replicates was flash frozen in liquid nitrogen for six taxa: *N. sylvestris*, *N. tomentosiformis*, *N. tabacum* 095-55, *N. tabacum* ‘Chulumani’, synthetic *N. tabacum* QM24, and synthetic *N. tabacum* QM25. Due to lack of material, only two biological replicates for *N. tomentosiformis* at 85% and QM25 at 95% were collected. The RNeasy Mini Plant Kit (Qiagen, Hilden, Germany) was used for RNA extraction, followed by DNase treatment using the Turbo-DNase Kit (Ambion, Vilnius, Lithuania). Strand-specific libraries were constructed from mRNA as previously described by [103] and sequenced on an Illumina HiSeq2500 (Illumina, San Diego, CA, USA) with 1 × 85 bp reads. Raw reads were processed using TrimGalore (version 0.4.2; [104]) to remove adapters and filter with a minimum quality score of 30 and a minimum length of 60 bp. 

Oxford Nanopore minION long reads were sequenced for stages 60% and 95% for both progenitor species. A total of 100 ng of total RNA was used as input for the cDNA-PCR Barcoding library kit (SQK-PCS109 with SQK-PBK004 barcodes). Four barcoded libraries were pooled with equal volumes. The loaded flowcell was run for 48 h, generating 5.86 million reads and 6.56 GB of raw data, followed by flushing (kit EXP-WSH003) and loading the remaining library for another 24 h, generating an additional 588 thousand reads and 656 MB of data. Fastq files were called from the produced fast5 files using guppy (version 3.4.1; Oxford Nanopore) with a minimum quality score of 7. Fastq reads were demultiplexed using porechop (version 0.2.4; https://github.com/rrwick/Porechop) and error corrected using FMLRC (version 1.0.0; [105]) by first building an index using the cffq function in MSBWT (version 0.3.0; [106]) from the Illumina sequencing reads. FMLRC has been shown to outperform other error correction approaches [107,108]. The resulting corrected reads were filtered with seqkit (version 0.12.0; [109]) to keep reads larger than 800 bp. Accession numbers to the Sequencing Read Archive for each accession are provided in Table S1.

A *de novo* transcriptome representing *N. tabacum* was necessary to map individual reads to characterize expression patterns because previous attempts to investigate differential gene expression using the reference genomes [110,111] resulted in a large number of unmapped reads. To minimize homeolog bias during assembly, a species level assembly for both *N. sylvestris* and *N. tomentosiformis* were merged in silico to represent *N. tabacum*. Multiple approaches were undertaken to find the most suitable combination of programs and reads to generate the most complete assemblies in terms of number of contigs, N50, and percent of Solanales Benchmarking Universal Single-Copy Orthologs (BUSCO; version 3.0.2; [112]). Assemblies were carried out with SPAdes (version 3.13.1; [113]), Trinity (version 2.8.4; [114,115]), and TransAbyss (version 2.2.1; [116]). Default parameters for SPAdes were employed. For TransAbyss, a kmer of 64 was used. Default parameters were used for Trinity with the exception of requiring a minimum contig length of 250 bp. Trinity and TransAbyss assemblies were done on the high-performance computing cluster at the University of California, Riverside, while the SPAdes assemblies were done on the CUPAC (Cornell University Plant Anatomy Collection) server. Additional Illumina data from previous studies were downloaded from the European Nucleotide Archive (ENA; Table S1) and processed with TrimGalore with a minimum quality of 30 and minimum length of 75 bp. Cleaned reads from each accession were normalized to 100× coverage using the BBNorm function in BBTools (version 38.16; http://jgi.doe.gov/data-and-tools/bbtools). The different combinations of data and programs were as follows: (1) SPAdes Illumina data, (2) SPAdes Illumina + ENA + Nanopore, (3) SPAdes ENA + Nanopore, (4) Trinity Illumina, (5) Trinity ENA + Nanopore, and (6) TransAbyss ENA + Nanopore. Assembly quality was estimated by calculating N50 and N90 values using the R package CNEr (version 1.18.1; [117]) after removing contigs smaller than 250 bp using seqkit (version 0.12.0). The percent of BUSCO genes in each assembly was determined using the Solanales library (solanaceae_odb10) with the transcriptome function. Plots were made in R (version 3.6.0; [99]) using the generate_plot.py script.

Filtered assemblies for *N. sylvestris* and *N. tomentosiformis* using ENA and Nanopore data were annotated using Trinotate [118] and TransDecoder (version 5.0.2; [115]). The assembly fasta file was prepped for alignment using RSEM (version 1.3.0; [119]) and bowtie2 (version 2.3.4.1; [120]). Contigs were compared to the SwissProt database with ncbi-blast (version 2.7.1+; [121] using BLASTX and BLASTP. The translated peptide assemblies were analyzed with OrthoVenn2 [122] to compare the number of overlapping proteins and the unique proteins in each species.

Differential gene expression was determined using limma (version 3.40.6; [123]) and Trimmed Mean of M values (TMM) normalization. Two sets of analyses were performed: one comparing 60%, 85%, and 95% of anthesis length developmental stages within a taxon and one comparing taxa at each developmental stage. The resulting BAM files were sorted and indexed using samtools (version 1.9; [124]) and visualized in IGV (version 2.5.3; [125,126,127]) to check for quality of mapping to candidate genes. Candidate genes were identified via the Trinotate annotation file. For known genes that were not annotated, we used a local BLAST (version 2.2.31) analysis using the TransDecoder CDS file with a minimum cutoff of length 50 bp and minimum e-value 1.0 × 10^−20^. Plots were generated to visualize differentially expressed genes using ggplot2. We plotted the log_2_ fold change of differentially expressed genes for each pairwise comparison using violin plots to represent all differentially expressed genes and overlaid strip plots representing the TMM differentially expressed values of organ size genes of interest. Clustering of differentially expressed transcripts was done in the Trinity pipeline with supplied perl scripts using hierarchical clustering of FPKM (Fragments Per Kilobase Million) expression values. 

## 3. Results

### 3.1. Cell width, Cell Number, and Ploidy Positively Influence Corolla Tube Circumference

**Figure 2:**
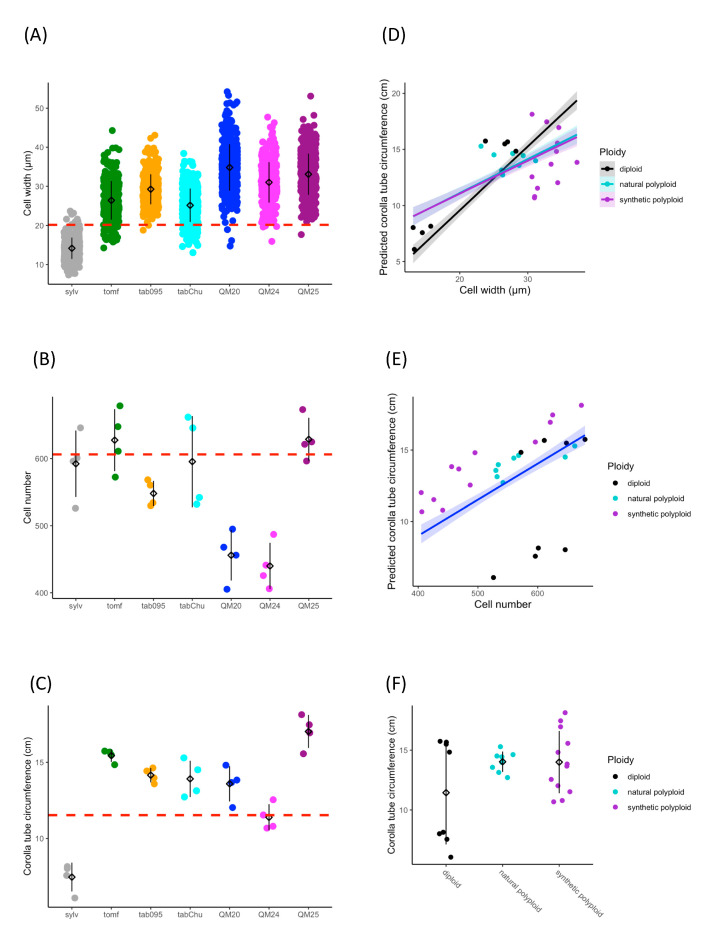
Strip plots for (**A**) cell width from 100 cells, (**B**) cell number, and (**C**) corolla tube circumference for all accessions, including mean and standard deviation for each accession: red dotted lines represent the progenitor average, which is the expected intermediate phenotype. Predicted corolla tube circumference based on the linear model for (**D**) cell width and (**E**) cell number: points represent actual flower data. Corolla tube circumference based on (**F**) ploidy including mean and standard deviation for each ploidy: points represent actual flower data. Sylv = *N. sylvestris*, tomf = *N. tomentosiformis*, tab095 = *N. tabacum* 095-55, tabChu = *N. tabacum* ‘Chulumani’, QM20 = synthetic *N. tabacum* QM20, QM24 = synthetic *N. tabacum* QM24, QM25 = synthetic *N. tabacum* QM25.

Based on previous studies showing wider corolla tubes in allopolyploids than expected [90,91], we tested the hypothesis that increases in tube width are due to increased cell width in polyploids. Cell size, cell number, and corolla tube circumference vary across accessions (Figure 2A–C). Ploidy positively influences cell width (*F*_2,25_ = 23.67, *p* = 1.71 × 10^−6^), natural polyploids have significantly wider cells than diploids (TukeyHSD, *p* = 0.006), and synthetic polyploids have significantly wider cells than both diploids (TukeyHSD, *p* = 1 × 10^−6^) and natural polyploids (TukeyHSD, *p* = 0.012; Figure 2A and Table 1). Ploidy also affects cell number (*F*_2,25_ = 4.892, *p* = 0.016), and synthetic polyploids have significantly fewer cells than diploids and natural polyploids (TukeyHSD, *p* = 0.015; Figure 2B and Table 2). We found that cell width (*F*_1,21_ = 181.47, *p* = 4.39 × 10^−14^), cell number (*F*_1,21_ = 240.12, *p* = 5.74 × 10^−13^), and ploidy (*F*_2,21_ = 6.56, *p* = 0.0061) all have a significant positive effect on corolla tube circumference by using a linear model (Figure 2D–F and Table 3; Table 4). Additionally, the interaction between cell width and ploidy is significant (*F*_2,21_ = 7.84, *p* = 0.0029; Table 4). This suggests that cell number affects corolla tube circumference independent of ploidy, but that the effect of cell width varies by ploidy. Both natural polyploids and synthetic polyploids have significantly less steep positive slopes for the effects of cell width on corolla tube circumference than diploids (*p* = 0.01 and *p* = 0.005, respectively; Figure 2D and Table 3), which implies that an increase in cell width in polyploids will not influence corolla tube circumference as much as an increase in cell width in diploids. However, natural polyploids show a marginal difference and synthetic polyploids have significantly larger corolla tube circumferences than diploids at 3rd quantile cell width values, and both natural and synthetic polyploids have a significantly larger corolla tube circumference than diploids at the maximum cell width value (Table S2). There is no difference in corolla tube circumference between diploids, natural polyploids, and synthetic polyploids at 1st quartile or median cell width values, whereas natural polyploids seem to have smaller corolla tube circumferences than diploids at the minimum cell width value (Table S2). In summary, cell width, ploidy, the interaction of width by ploidy, and cell number are all crucial for determining corolla tube circumference (Table 4).

### 3.2. Transcriptome Assemblies

Nanopore sequencing generated 5,380,172 high-quality reads. Specifically, 1,728,564 reads were generated for *N. sylvestris* 60% and 995,999 reads were generated at 95%. For *N. tomentosiformis*, there were 992,903 reads generated at 60% and 1,662,706 reads generated at 95%. After removing reads shorter than 800 bp, a total of 1,058,332 reads for *N. sylvestris* with a mean read length of 1171 bp and 952,350 reads for *N. tomentosiformis* with a mean read length of 1150 bp remained.

The number of assembled contigs, associated N50 values, and represented BUSCO genes varies greatly among the different assemblies. The only combination that failed to assemble was Trinity using ENA + Nanopore with the --long_reads option. For *N. sylvestris*, in terms of the size and number of contigs, the best assembly was with SPAdes using ENA + Nanopore. This assembly had 37,710 contigs with a N50 of 2356 bp and N90 of 788 bp (Table 5). Using just Illumina data generated an assembly with 44,350 contigs and an N50 of 1716 bp. Combining all data generated 39,620 contigs and a N50 of 2254 bp. The assembly with all data was slightly worse than ENA + Nanopore with close to 2500 additional contigs and a 100 bp smaller N50 value; even though there were more total reads, the Illumina data from developmental stages were short (85 bp) and only single-end. The worst assemblies were the Trinity assembly (119,790 contigs, N50 of 815) and the TransAbyss assembly with ENA + Nanopore (100,463 contigs, N50 of 1172).

A similar pattern of quality of assembly was seen for *N. tomentosiformis* with the SPAdes ENA + Nanopore assembly producing 39,950 contigs, a N50 of 2258 bp, and a N90 of 643 bp. The second-best assembly was the SPAdes Illumina only, with the SPAdes Illumina + ENA + Nanopore generating a much worse assembly in terms of number of contigs (51,360 contigs vs. 47,568 contigs) but a better N50 value (2258 bp vs. 1704 bp). Again, the worst assemblies were the Trinity Illumina and the TransAbyss assemblies.

**Figure 3:**
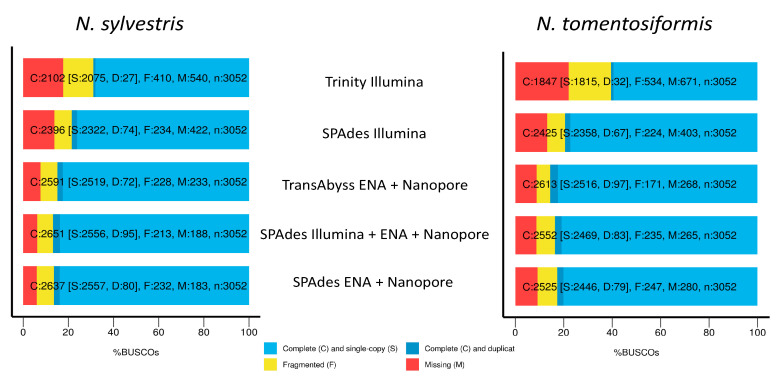
Percent complete single copy, fragmented, and missing BUSCO genes for both *N. sylvestris* and *N. tomentosiformis* across the five different assemblies performed: including the Nanopore long-reads gave an increase of complete single copy genes and decrease in missing genes compared to short-read only, regardless of assembler.

For completeness in terms of percent BUSCO genes present for *N. sylvestris*, the best assemblies were SPAdes Illumina + ENA + Nanopore, SPAdes ENA + Nanopore, and TransAbyss. For complete single-copy genes, all three ranged from 84.9 to 86.8%, with 6–7.6% genes missing. The Trinity assembly and SPAdes Illumina assemblies had the lowest percentage of complete single copy genes, the largest percentage of fragmented genes, and the largest percentage of missing genes (Figure 3 and Table 5). For *N. tomentosiformis*, the best assemblies for complete single-copy genes are the same, but with the TransAbyss assembly having slightly higher percentage of complete single-copy genes and lower fragmented genes. The Trinity and SPAdes Illumina assemblies were the worst, with the Trinity assembly coming in far below the others in terms of complete single-copy genes and over double the amount of fragmented and missing genes.

We used the SPAdes ENA + Nanopore assemblies for further analyses, and these assemblies have a total of 6115 shared protein clusters for both progenitor species, with an additional 460 protein clusters unique to *N. tomentosiformis* and 480 protein clusters unique to *N. sylvestris* (Figure S2 and Table S3).

### 3.3. Differential Expression

Through development, there is a pattern in which the 85% and 95% stages are most similar based on gene expression (Figure S3) while the most differentially expressed (DE) genes occur in comparisons between 60% and 95% (Table 6). Within *N. sylvestris*, of the total 37,097 transcripts, there are 35 DE transcripts between 60% and 85%, 537 DE transcripts between 60% and 95%, and zero DE transcripts between 85% and 95% using a cutoff of False Discover Rate (FDR) = 0.05 and a 4-fold change in expression (Table 6). Within *N. tomentosiformis*, of the 39,950 transcripts, there are 227 DE transcripts between 60% and 85%, 801 DE transcripts between 60% and 95%, and no DE transcripts between 85% and 95%. For the natural polyploids *N. tabacum* ‘Chulumani’ and 095-55, the comparisons with the most DE transcripts are between 60% and 95% with 4289 and 3796, respectively. In comparison, the synthetic *N. tabacum* accessions QM24 and QM25 show 2914 and 2720 transcripts differentially expressed.

When looking at clustering of expression patterns across all taxa, there is a cluster of natural polyploids and a cluster of synthetic polyploids, with the two progenitor species being quite distinct from all polyploids (Figure S4). In fact, based on patterns of DE transcripts, *N. tomentosiformis* appears to be more similar to all *N. tabacum* accessions at 60% and 95% while *N. sylvestris* is more similar to the *N. tabacum* accessions at 85%. At 60%, we see 43,147 DE transcripts between the progenitor species. Between the two natural polyploids, there are 781 DE transcripts, and between the two synthetic polyploids, there are 177 DE transcripts. The same pattern exists at 85% with 42,275 DE transcripts between progenitor lines, 605 DE transcripts between the natural polyploids, and 228 DE transcripts between the synthetic polyploids. Within the 95% comparisons, there are 479 DE transcripts between natural polyploids but no differentially expressed transcripts between the synthetic polyploids. Between the diploid progenitors at 95%, there are 43,023 DE transcripts.

**Figure 4:**
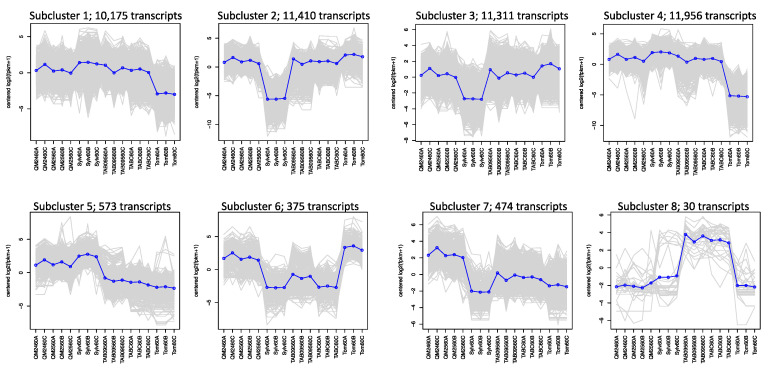
Subclustering of transcripts across all taxa within the 60% of anthesis developmental stage based on hierarchical clustering of differentially expressed genes: the number of transcripts found in each cluster is given. Each gray line is one gene, while the blue line is the mean expression profile for that subcluster. The order along the x-axis for each subcluster plot is synthetic polyploids (QM24 and QM25), *N. sylvestris* (Sylv), natural polyploids (TAB095 and TABC), and *N. tomentosiformis* (Tom).

Hierarchical clustering based on differential expression for the 60% comparisons yielded 8 subclusters (Figure 4). In the first four subclusters, representing 44,852 transcripts, both natural and synthetic polyploids show expression patterns similar to one or the other progenitor. Subcluster 5 with 573 transcripts shows that the synthetic polyploids and *N. sylvestris* have similar expression patterns whereas the natural polyploids and *N. tomentosiformis* have similar expression patterns. Subcluster 6 with 375 transcripts shows the opposite pattern, with the synthetic lines similar to *N. tomentosiformis* and the natural polyploids similar to *N. sylvestris*. The remaining two clusters show transgressive expression patterns with the synthetic polyploids and natural polyploids showing the highest expression in subcluster 7 (474 transcripts) and subcluster 8 (30 transcripts), respectively. The breakdown of annotated transcripts in each subcluster is presented in Table S4.

For comparisons within the 85% developmental stage, 12 subclusters were identified. Five subclusters comprising 42,080 transcripts show a pattern with the polyploids being similar to one of the progenitors (Figure S5). Subcluster 4 (353 transcripts) and subcluster 7 (646 transcripts) show similar expressions between the synthetic lines and *N. sylvestris*, whereas the natural polyploids resemble *N. tomentosiformis*. Subcluster 12 (18 transcripts) shows the opposite pattern, with the natural polyploids similar to *N. sylvestris* and the synthetic lines similar to *N. tomentosiformis*. Subcluster 8 (520 transcripts) shows the synthetic polyploids displaying transgressive upregulated expression. Three subclusters show unique patterns not found in the 60% developmental stage. In subcluster 5 (1986 transcripts), both the synthetic and natural polyploids appear to have an intermediate expression between the two progenitor species. Subcluster 9 (215 transcripts) shows that the natural polyploid *N. tabacum* 095-55 is similar to *N. tomentosiformis*, whereas *N. tabacum* ‘Chulumani’ is similar to *N. sylvestris*. Subcluster 10 (122 transcripts) shows that *N. tabacum* 095-55 is similar to *N. sylvestris*, whereas *N. tabacum* ‘Chulumani’ is intermediate between the two progenitors. The breakdown of annotated contigs in each subcluster is presented in Table S5.

The comparisons within the 95% development stage showed 11 subclusters (Figure S6). Three subclusters representing 32,189 transcripts show both polyploids similar to one of the progenitor lines. Three subclusters show transgressive expression in the polyploids. In subcluster 5 (2405 transcripts), both synthetic and natural polyploids have higher expression than the progenitors. In subcluster 8 (185 transcripts), the natural polyploids have higher expression, and in subcluster 9 (93 transcripts), the synthetic lines have higher expression. Subclusters 7 (254 transcripts) and 10 (122 transcripts) show that the synthetic lines and *N. tomentosiformis* have similar expression profiles, as do the natural polyploids and *N. sylvestris*. Subcluster 11 (40 transcripts) has similar expression between synthetic lines and *N. sylvestris*, whereas the natural polyploids are similar to *N. tomentosiformis*. The final two clusters, subcluster 3 (10,385 transcripts) and subcluster 6 (885 transcripts), show both natural and synthetic polyploids with intermediate expression levels between the two progenitors. The breakdown of annotated contigs in each subcluster are presented in Table S6.

**Figure 5:**
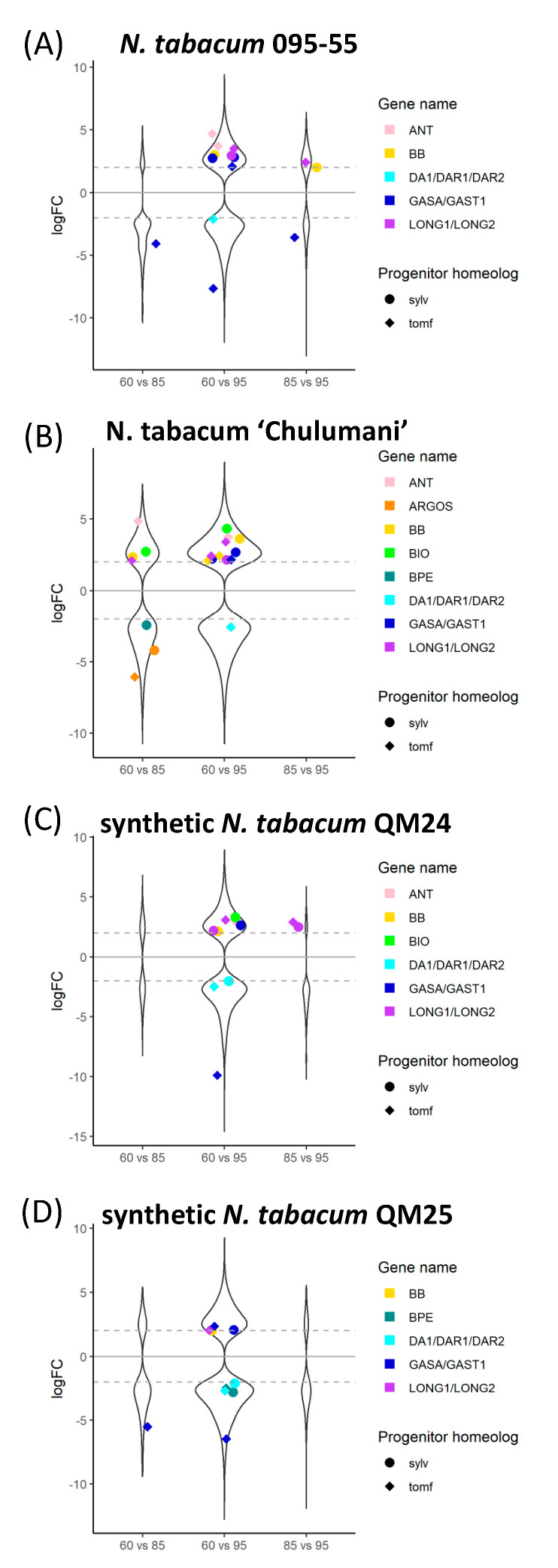
Log_2_ fold change of differentially expressed genes across comparisons within accessions between 60%, 85%, and 95% developmental time points for (**A**) *N. tabacum* 095-55, (**B**) *N. tabacum* ‘Chulumani’, (**C**) synthetic *N. tabacum* QM24, and (**D**) synthetic *N. tabacum* QM25: violin plots represent all differentially expressed genes between each comparison, whereas colorful strip plots represent differentially expressed candidate floral organ size genes. Positive logFC values represent genes upregulated in the first stage listed in the comparison, whereas negative logFC values represent genes upregulated in the second stage listed. The dashed gray lines across the plot denote the log_2_ = |2| cutoff for differentially expressed genes. Sylv = *N. sylvestris*, tomf = *N. tomentosiformis*.

We were able to identify many of the known candidate genes involved in floral organ size. In general, we see more candidate genes differentially expressed within the different polyploid accessions in comparisons between 60% and 95% (Figure 5); however, we do see differential expression within polyploid accessions in at least a few genes across all stages. A few candidate genes show differential expression within *N. sylvestris* or *N. tomentosiformis* through development except for *BPE* which is upregulated in 95% in *N. sylvestris* compared to 60% (Figure S7). As expected, comparisons between polyploids and one of their diploid progenitors show that polyploids display upregulation of the other progenitor homeolog (Figure S8). The *N. sylvestris* copy of *GASA/GAST1* appears to have higher levels of expression in all polyploids compared to the expression found within *N. sylvestris* across all developmental stages (Figure S8). We also see downregulation of some of the *N. sylvestris* genes that act as inhibitors, such as *BIG BROTHER (BB)* and *BIGGER ORGANS*, in the polyploids compared to expression in *N. sylvestris* as well as downregulation of the *N. tomentosiformis* copy of *BB* in polyploids compared to expression in *N. tomentosiformis* (Figure S8).

**Figure 6:**
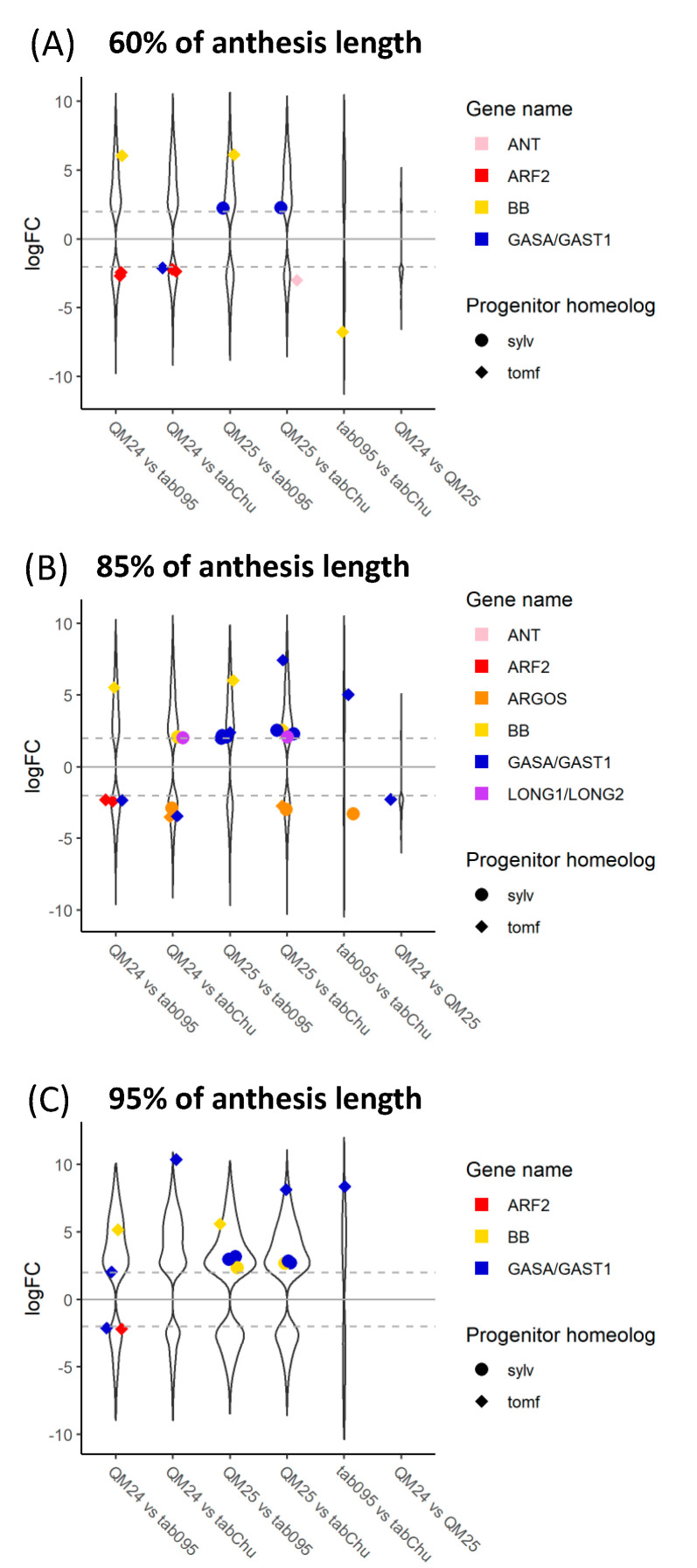
Log_2_ fold change of differentially expressed genes across comparisons between natural and synthetic *N. tabacum* accessions at (**A**) 60%, (**B**) 85%, and (**C**) 95%of anthesis length: violin plots represent all differentially expressed genes between each comparison, whereas colorful strip plots represent differentially expressed candidate floral organ size genes. Positive logFC values represent genes upregulated in the first accession listed in the comparison, whereas negative logFC values represent genes upregulated in the second accession listed. The dashed gray lines across the plot denote the log_2_ = |2| cutoff for differentially expressed genes. Sylv = *N. sylvestris*, tomf = *N. tomentosiformis*, tab095 = *N. tabacum* 095-55, tabChu = *N. tabacum* ‘Chulumani’, QM24 = synthetic *N. tabacum* QM24, and QM25 = synthetic *N. tabacum* QM25.

Comparing the natural and synthetic polyploids at 60%, we see downregulation of the *N. tomentosiformis* copy of *BB* in *N. tabacum* 095-55 compared to all other polyploids (Figure 6). For QM24, the *N. tomentosiformis* copy of *ARF2* appears to be downregulated compared to the two natural polyploids, whereas the transcripts in QM25 do not show differences. The *N. sylvestris* copy of *GASA/GAST1* appears to be upregulated in QM25 compared to the natural polyploids, whereas the transcript in QM24 does not show any difference.

In the comparisons at 85%, we see upregulation of both progenitor copies of *ARGOS* in the natural polyploid *N. tabacum* ‘Chulumani’. The *N. tomentosiformis* copies of *GASA/GAST1* appear downregulated in QM24 compared to the natural polyploids and QM25, whereas copies from both progenitors appear upregulated in QM25 compared to both natural polyploids. We also see upregulation of the *N. tomentosiformis* copy of *BB* in both QM24 and QM25 compared to *N. tabacum* 095-55, whereas *BB* from *N. sylvestris* is upregulated in both QM24 and QM25 compared to *N. tabacum* ‘Chulumani’. Additionally, the *LONGIFOLIA* genes from *N. sylvestris* appear to be upregulated in both synthetic lines compared to *N. tabacum* ‘Chulumani’ but show no expression differences with *N. tabacum* 095-55.

At 95%, we see consistent upregulation of the *N. tomentosiformis* copy of *GASA/GAST1* in QM24 compared to natural polyploids (we also see upregulation in QM25 versus *N. tabacum* ‘Chulumani’), while in QM25, the *N. sylvestris* copies of *GASA/GAST1* show upregulation compared to the natural polyploids. We generally see upregulation of *BB* in synthetic lines compared to the natural polyploids (except in QM24 compared to *N. tabacum* ‘Chulumani’).

## 4. Discussion

*Nicotiana* polyploids have significantly wider cells than diploids, with synthetic polyploids having even wider cells than natural polyploids, and the ploidy factor in our model, which includes three levels—diploids, natural polyploids, and synthetic polyploids—significantly increases corolla tube circumference. Synthetic polyploids show a decrease in the number of differentially expressed transcripts within a taxon through development compared to natural polyploids. Clustering by expression patterns shows a relatively small number of transcripts displaying transgressive expression in either the natural or synthetic polyploids compared to the progenitors, while even fewer transcripts show an intermediate expression profile. Many of the known candidate genes for floral organ size show differential expression, specifically *ARF2*, *BB*, and *GASA/GAST1*.

Liqin et al. [49] showed upregulation of *BB* in diploids compared to synthetic triploids and synthetic tetraploids of *Populus* and suggested that *BB* acts as a species-specific organ size checkpoint. More importantly, Liqin et al. [49] put forward the hypothesis that polyploids may need higher expression levels of *BB* to obtain normal organ size. *BIG BROTHER* was originally described as limiting organ size by restricting the period of proliferative growth [26]. Here, we see higher expression levels of the *N. tomentosiformis* copy across all three developmental time points in the synthetic polyploids than the natural polyploids. In addition to upregulation of *BB* compared to other *N. tabacum* accessions, the synthetic polyploid QM25, which has the largest flowers of all the polyploids investigated, shows higher expression of *GASA/GAST1* than the natural polyploids, especially the copy from *N. sylvestris*. Members of the *GASA* family, in particular *GASA4*, are expressed in developing roots and flowers [24]. Indeed, in *Saltugilia* (Polemoniaceae), *GASA* was expressed at the highest levels at mature stages, which corresponded to cell elongation in the petal tube [50,128]. The accession QM25 has the longest corolla tube examined excluding *N. sylvestris* (Figure 1). The interaction between a gene that represses proliferation (such as *BB* [26]) and a gene that promotes cellular elongation (such as *GASA* [24]) in polyploids is intriguing, especially since the upregulated homeolog of the repressor is from the shorter flowered progenitor and the upregulated homeolog that promotes elongation comes from the longer flowered progenitor. In addition, the *N. tomentosiformis* copy of *ARF2*, which is involved in promoting cell proliferation [34], is downregulated in QM24 compared to the natural polyploids; this downregulation may play a role in restricting cell division in this accession because QM24 has the fewest cells of the accessions examined (Figure 2B).

### 4.1. Differences in Expression

We see that natural and synthetic polyploids have expression patterns similar to one progenitor for the vast majority of transcripts across all developmental stages (Figure 4 and Figures S5 and S6). In total, there are 1612 transcripts in which the synthetic lines and *N. sylvestris* share similar expression patterns, whereas the natural polyploids are similar to *N. tomentosiformis*. Conversely, there are only 769 transcripts where the synthetic lines have similar expression to *N. tomentosiformis*, whereas the natural polyploids were similar to *N. sylvestris.* This demonstrates that the expression patterns in the synthetic lines tend to more closely resemble those of the maternal progenitor, *N. sylvestris*, whereas those in the natural polyploids tend to be more like the paternal progenitor, *N. tomentosiformis*.

The scaling of transcription with cell size has been shown previously [129,130,131], yet gene expression scales independently of ploidy [132,133] with no universal linear relationship [134]. We see distinct differences in cell width between natural and synthetic polyploids (Figure 2) and that natural and synthetic polyploids form two distinct clusters based on patterns of differentially expressed genes across developmental stages. Even with the same genomic input, we see clear evidence at the phenotypic and transcriptomic levels that the two types of polyploids are distinctly different, with clear differences in floral size (Figure 1).

### 4.2. De Novo Transcriptome Assembly

Before undertaking the *de novo* approach, we tried appropriate analyses using the published genomes currently available for both progenitor species, *N. sylvestris* and *N. tomentosiformis* [110], as well as for *N. tabacum* [111]. Likely due to the total number of contigs in each assembly (253,917 contigs for *N. sylvestris*, 159,598 contigs for *N. tomentosiformis*, and 1,084,432 contigs for *N. tabacum*) and possible fragmentation of genes, we were unable to get clear results for differentially expressed genes, including genes associated with the anthocyanin pathway [135], which based on our sampling of developmental stage should have shown differential expression. The generated *de novo* assemblies were only slightly worse in regards to missing data than the published genomes with 6% and 9.2% missing BUSCO genes compared to 2.2% and 2.5%, which provided the power to characterize differential expression at a finer scale than just being able to characterize the broadest gene ontology terms [136]. The *de novo* assemblies proved better than the assembled genomes with fewer fragmented contigs of candidate genes of organ size and flower color. Similar to other recent studies, there is a wide distribution of quality of assemblies based on the programs and methods used [137,138]; in our case, we saw that SPAdes outperformed Trinity in regards to completeness of BUSCO genes and number of contigs. Adding just over a million long-reads to the *N. sylvestris* transcriptome decreased the number of contigs by nearly 7000 and increased the N50 values of the assembly by approximately 500 bp while increasing the complete single copy BUSCO genes from 78.5% to 86.4% (Figure 3). Similarly, for *N. tomentosiformis*, adding 950 thousand long-reads decreased the number of contigs by over 7700, increased the N50 from 1704 bp to 2365 bp, and increased the percent complete single copy genes from 79.5% to 82.7%. The addition of long-reads allowed for differentiation between *N. sylvestris* and *N. tomentosiformis* homeologs, which was not possible with only the Illumina data [139]. 

### 4.3. Cell Size Increase Results in Wider Corolla Tubes

Young allopolyploids tend to evolve shorter and wider corolla tubes than expected based on the mean of the morphology of their progenitors, which may allow for a greater variety of pollinator types to access the nectar rewards of these flowers [90,91]. Cell width, cell number, and ploidy positively influence corolla tube circumference, and polyploids have significantly wider cells than diploids, with either the same number of cells or fewer (Figure 2). Thus, the wider tubes in allopolyploids result largely from an increase in cell width. The increase of cell size may result from increased growth during longer cell cycle times associated with increased genome content [140].

Other studies have also found a correlation between higher ploidy and increased cell size [59,63,64,65,141]. These results suggest that an increase in cell size may be a direct consequence of the increase in genome size that is inherent in polyploidization [58]. Further evidence from *Arabidopsis thaliana* diploid, triploid, and tetraploid hybrids indicate that guard cell size increases with ploidy but that hybrids of the same ploidy as their progenitors do not display an increase [142]. Therefore, the increase in cell size is driven by polyploidy, not hybridization. Thus, the wider tubes observed in young *Nicotiana* polyploids may be a direct result of increased cell width at polyploid origin due to genome duplication. Similarly, flower size increased with polyploidy in colchicine-induced autotetraploids of pomegranate (*Punica granatum*; [143], *Phlox* [144], and *Gerbera* [145]; in natural autopolyploid populations [146]; and in synthetic allopolyploid lines of *A. thaliana* [142,146] and *Brassica napus* [147].

Intriguingly, synthetic allopolyploids have significantly wider cells than naturally occurring allopolyploids that arose approximately 0.6 million years ago [87]. This suggests that increased cell size in polyploids may attenuate over time [71], which correlates with our results that young polyploids tend to have wider corolla tubes than expected while older polyploids do not [90]. However, this attenuation of cell size in natural polyploids does not seem to be due solely to reduction of genome size following polyploidy because the natural *N. tabacum* genome has only downsized 3.7% compared to the expected sum of its progenitors [148]. In addition, these cell size results are from a single allopolyploid species represented by only two natural accessions and three synthetic lines. Another possibility is that the colchicine treatment and tissue culture processes used in the creation of the synthetic lines has affected cell size in the synthetic allopolyploids. However, a study separating the effects of polyploidy and colchicine treatment found that guard cell length was primarily affected by ploidy [149]. In addition, conical cells in floral limb tissue of *Nicotiana rustica* (approximately 0.7 million years old) are nearly the sum of those of its progenitor species while conical cells of three species of *Nicotiana* section *Repandae* (approximately 4.3 million years old) [87] are intermediate in area between those of their progenitors [150]. This intermediate cell size is consistent with the fact that tube width in section *Repandae* is intermediate between progenitor morphology, as expected [91]. The potential reversion to a diploid-like cell size over time may explain why older polyploids do not display the same trends in corolla tube width evolution as young polyploids [90]. Again, this reversion to a diploid-like cell size does not seem to be correlated to genome size because two of the section *Repandae* species examined have genome sizes that have increased following polyploidy based on the sum of their diploid progenitors [148].

## 5. Conclusions

Inclusion of Oxford Nanopore long-read data significantly improved our transcriptome assemblies such that they contained a higher percentage of BUSCO genes and allowed for differentiation between progenitor homeologs. Floral organ size genes *ARF2*, *BIG BROTHER*, and *GASA/GAST1* show upregulation in natural and synthetic *N. tabacum* accessions compared to the progenitor species. The synthetic polyploid accessions also showed upregulation of the candidate genes compared to the natural polyploids. The highest expression of candidate genes was observed in earlier stages of flower development. The wider corolla tubes observed in young allopolyploids are predominantly due to an increase in cell width, perhaps resulting from whole genome duplication, which suggests that morphological change toward more generalist pollination may be a direct consequence of polyploidy.

## Figures and Tables

**Table 1:** Linear model results for width~ploidy.

Coefficients	Estimate	SE	*t* Value	*p* Value	Tukey HSD	*p* Value
Intercept	20.29	1.43	14.20	1.8 × 10^−13^	diploid-natural polyploid	0.0059
Ploidy-natural polyploid	6.91	2.02	3.42	0.00217	diploid-synthetic polyploid	1.0 × 10^−6^
Ploidy-synthetic polyploid	12.67	1.85	6.87	3.37 × 10^−7^	natural polyploid-synthetic polyploid	0.012

**Table 2:** Linear model results for cell number~ploidy.

Coefficients	Estimate	SE	*t* Value	*p* Value	Tukey HSD	*p* Value
Intercept	609.88	25.91	23.53	2 × 10^−16^	diploid-natural polyploid	0.0059
Ploidy-natural polyploid	−38.13	36.65	−1.04	0.30812	diploid-synthetic polyploid	1.0 × 10^−6^
Ploidy-synthetic polyploid	−101.63	33.45	−3.04	0.00551	natural polyploid-synthetic polyploid	0.012

**Table 3:** Linear model results for corolla tube circumference~width + ploidy + width: ploidy + cell number.

Coefficients	Estimate	SE	*t* Value	*p* Value
Intercept	−15.47	1.15	−13.47	8.39 × 10^−12^
Width	0.57	0.03	17.67	4.39 × 10^−14^
Ploidy-natural polyploid	6.86	2.48	2.76	0.01164
Ploidy-synthetic polyploid	7.01	2.81	2.50	0.02097
Cell number	0.03	0.002	15.50	5.74 × 10^−13^
Width:ploidy-natural polyploid	−0.27	0.09	−2.83	0.00995
Width:ploidy-synthetic polyploid	−0.28	0.09	−3.12	0.00516

**Table 4:** ANOVA results for corolla tube circumference~width + ploidy + width: ploidy + cell number.

Coefficients	Sum Sq	DF	*F* Value	*p* Value
Intercept	58.68	1	181.47	8.39 × 10^−12^
Width	100.98	1	312.27	4.39 × 10^−14^
Ploidy	4.24	2	6.56	0.0061
Cell number	77.65	1	240.12	5.74 × 10^−13^
Width:ploidy	5.07	2	7.84	0.0029
Residuals	6.79	21		

**Table 5:** Assembly statistics for each transcriptome assembly performed for *N. sylvestris* and *N. tomentosiformis* including the assembler used, data that went into the assembly, number of contigs, N50, N90, percent complete single-copy BUSCO genes, percent fragmented BUSCO genes, and percent missing BUSCO genes.

***Nicotiana sylvestris***							
**Assembler**	**Data**	**Number of Contigs**	**N50**	**N90**	**% Complete Single Copy**	**% Fragmented**	**% Missing**
SPAdes	Illumina	44,350	1716	463	78.5	7.7	13.8
SPAdes	Illumina + ENA + nanopore	39,620	2254	760	86.8	7	6.2
SPAdes	ENA + nanopore	37,097	2356	788	86.4	7.6	6
Trinity	Illumina	119,790	815	336	68.9	13.4	17.7
Trinity	ENA + nanopore *	NA	NA	NA	NA	NA	NA
TransAbyss	ENA + nanopore	100,463	1172	357	84.9	7.5	7.6
***Nicotiana tomentosiformis***							
**Assembler**	**Data**	**Number of Contigs**	**N50**	**N90**	**% Complete Single Copy**	**% Fragmented**	**% Missing**
SPAdes	Illumina	47,658	1704	416	79.5	7.3	13.2
SPAdes	Illumina + ENA + nanopore	51,360	2258	643	83.6	7.7	8.7
SPAdes	ENA + nanopore	39,950	2365	777	82.7	8.1	9.2
Trinity	Illumina	132,048	788	337	60.5	17.5	22
Trinity	ENA + nanopore *	NA	NA	NA	NA	NA	NA
TransAbyss	ENA + nanopore	129,220	1068	354	85.6	5.6	8.8

* Trinity assembly with the ENA + nanopore data failed to produce an output.

**Table 6:** Number of differentially expressed contigs within a taxon through the three developmental stages: 60% of anthesis, 85% of anthesis, and 95% of anthesis. The number of differentially expressed contigs with a 4-fold change in expression and FDR less than 0.05 are reported, along with the total number of transcripts present in the given comparison.

Taxon	60% vs. 85%	60% vs. 95%	85% vs. 95%
*N. sylvestris*	35/23,952	537/23,952	0/24,063
*N. tomentosiformis*	227/24,585	801/24,871	0/24,445
*N. tabacum* 095-55	860/43,263	3796/44,207	691/43,615
*N. tabacum* ‘Chulumani’	2241/43,638	4289/43,798	1/42,877
synthetic *N. tabacum* QM24	363/42,735	2914/42,879	262/42,937
synthetic *N. tabacum* QM25	679/43,416	2720/43,977	335/43,373

## Data Availability

SRA numbers for raw sequencing data files are found in Table S1. Cell size images have been uploaded to Figshare (https://doi.org/10.6084/m9.figshare.12499463). Code for cell analyses can be found on GitHub https://github.com/elizabethwmccarthy/.

## Supplementary Materials

The following are available online at https://www.mdpi.com/2073-4425/11/9/1097/s1. Figure S1: Diagram of corolla tube sampling, Figure S2: OrthoVenn diagram comparisons of protein clusters between progenitor transcriptomes, Figure S3: Hierarchical cluster of gene expression between developmental stages with a taxon, Figure S4: Hierarchical cluster of gene expression between taxa within a developmental stage, Figure S5: Subclustering of transcripts across all taxa at 85% anthesis, Figure S6: Subclustering of transcripts across all taxa at 95% anthesis, Figure S7: Differentially expressed candidate genes within each progenitor species at different developmental stages, Figure S8: Differentially expressed candidate genes between all comparisons at different developmental stages, Table S1: Sequencing information for each accession, Table S2: Pairwise comparisons of ploidy categories, Table S3: Protein comparison information between progenitor species, Table S4: Contigs for each subcluster at 60% anthesis, Table S5: Contigs for each subcluster at 85% anthesis, Table S6: Contigs for each subcluster at 95% anthesis.

## References

[1] Mizukami Y. (2001). A matter of size: Developmental control of organ size in plants. Curr. Opin. Plant Biol..

[2] Breuninger H., Lenhard M. (2010). Control of tissue and organ growth in plants. Curr. Top. Dev. Biol..

[3] Irish V.F. (2008). The Arabidopsis petal: A model for plant organogenesis. Trends Plant Sci..

[4] Hepworth J., Lenhard M. (2014). Regulation of plant lateral-organ growth by modulating cell number and size. Curr. Opin. Plant Biol..

[5] Kaplan D.R., Hagemann W. (1991). The Relationship of Cell and Organism in Vascular Plants. Bioscience.

[6] Feng G., Qin Z., Yan J., Zhang X., Hu Y. (2011). Arabidopsis organ size related1 regulates organ growth and final organ size in orchestration with ARGOS and ARL. New Phytol..

[7] Varaud E., Brioudes F., Szécsi J., Leroux J., Brown S., Perrot-Rechenmann C., Bendahmane M. (2011). AUXIN RESPONSE FACTOR8 regulates Arabidopsis petal growth by interacting with the bHLH transcription factor BIGPETALp. Plant Cell.

[8] Xu R., Li Y. (2011). Control of final organ size by Mediator complex subunit 25 in Arabidopsis thaliana. Development.

[9] Martin C., Gerats T. (1993). Control of Pigment Biosynthesis Genes during Petal Development. Plant Cell.

[10] Stuurman J., Hoballah M.E., Broger L., Moore J., Basten C., Kuhlemeier C. (2004). Dissection of floral pollination syndromes in Petunia. Genetics.

[11] Fenster C.B., Armbruster W.S., Wilson P., Dudash M.R., Thomson J.D. (2004). Pollination Syndromes and Floral Specialization. Annu. Rev. Ecol. Evol. Syst..

[12] Sargent R.D., Goodwillie C., Kalisz S., Ree R.H. (2007). Phylogenetic evidence for a flower size and number trade-off. Am. J. Bot..

[13] Goodwillie C., Sargent R.D., Eckert C.G., Elle E., Geber M.A., Johnston M.O., Kalisz S., Moeller D.A., Ree R.H., Vallejo-Marin M. (2010). Correlated evolution of mating system and floral display traits in flowering plants and its implications for the distribution of mating system variation. New Phytol..

[14] Moyroud E., Glover B.J. (2017). The Evolution of Diverse Floral Morphologies. Curr. Biol..

[15] Eckhart V.M. (1991). The effects of floral display on pollinator visitation vary among populations ofPhacelia linearis (Hydrophyllaceae). Evol. Ecol..

[16] Kettle C.J., Maycock C.R., Ghazoul J., Hollingsworth P.M., Khoo E., Sukri R.S.H., Burslem D.F.R.P. (2011). Ecological implications of a flower size/number trade-off in tropical forest trees. PLoS ONE.

[17] Solís-Montero L., Vallejo-Marín M. (2017). Does the morphological fit between flowers and pollinators affect pollen deposition? An experimental test in a buzz-pollinated species with anther dimorphism. Ecol. Evol..

[18] Krizek B.A., Anderson J.T. (2013). Control of flower size. J. Exp. Bot..

[19] Stebbins G.L. (1974). Flowering Plants: Evolution above the Species Level.

[20] Glover B.J. (2007). Understanding Flowers and Flowering: An Integrated Approach.

[21] Ojeda I., Santos-Guerra A., Caujapé-Castells J., Jaén-Molina R., Marrero Á., Cronk Q.C.B. (2012). Comparative Micromorphology of Petals in Macaronesian Lotus (Leguminosae) Reveals a Loss of Papillose Conical Cells during the Evolution of Bird Pollination. Int. J. Plant Sci..

[22] Krizek B.A. (2009). Making bigger plants: Key regulators of final organ size. Curr. Opin. Plant Biol..

[23] Shi L., Gast R.T., Gopalraj M., Olszewski N.E. (1992). Characterization of a shoot-specific, GA3- and ABA-regulated gene from tomato. Plant J..

[24] Herzog M., Dorne A.M., Grellet F. (1995). GASA, a gibberellin-regulated gene family from Arabidopsis thaliana related to the tomato GAST1 gene. Plant Mol. Biol..

[25] Kotilainen M., Helariutta Y., Mehto M., Pollanen E., Albert V.A., Elomaa P., Teeri T.H. (1999). GEG participates in the regulation of cell and organ shape during corolla and carpel development in gerbera hybrida. Plant Cell.

[26] Disch S., Anastasiou E., Sharma V.K., Laux T., Fletcher J.C., Lenhard M. (2006). The E3 Ubiquitin Ligase BIG BROTHER Controls Arabidopsis Organ Size in a Dosage-Dependent Manner. Curr. Biol..

[27] Szécsi J., Joly C., Bordji K., Varaud E., Cock J.M., Dumas C., Bendahmane M. (2006). BIGPETALp, a bHLH transcription factor is involved in the control of Arabidopsis petal size. EMBO J..

[28] Lee Y.K., Kim G.-T., Kim I.-J., Park J., Kwak S.-S., Choi G., Chung W.-I. (2006). LONGIFOLIA1 and LONGIFOLIA2, two homologous genes, regulate longitudinal cell elongation in Arabidopsis. Development.

[29] Kim G.-T., Shoda K., Tsuge T., Cho K.-H., Uchimiya H., Yokoyama R., Nishitani K., Tsukaya H. (2002). The ANGUSTIFOLIA gene of Arabidopsis, a plant CtBP gene, regulates leaf-cell expansion, the arrangement of cortical microtubules in leaf cells and expression of a gene involved in cell-wall formation. EMBO J..

[30] Hu Y., Xie Q., Chua N.-H. (2003). The Arabidopsis auxin-inducible gene ARGOS controls lateral organ size. Plant Cell.

[31] Guo M., Rupe M.A., Wei J., Winkler C., Goncalves-Butruille M., Weers B.P., Cerwick S.F., Dieter J.A., Duncan K.E., Howard R.J. (2014). Maize ARGOS1 (ZAR1) transgenic alleles increase hybrid maize yield. J. Exp. Bot..

[32] Krizek B. (2009). AINTEGUMENTA and AINTEGUMENTA-LIKE6 act redundantly to regulate Arabidopsis floral growth and patterning. Plant Physiol..

[33] Krizek B.A., Eaddy M. (2012). AINTEGUMENTA-LIKE6 regulates cellular differentiation in flowers. Plant Mol. Biol..

[34] Ellis C.M., Nagpal P., Young J.C., Hagen G., Guilfoyle T.J., Reed J.W. (2005). AUXIN RESPONSE FACTOR1 and AUXIN RESPONSE FACTOR2 regulate senescence and floral organ abscission in Arabidopsis thaliana. Development.

[35] Anastasiou E., Kenz S., Gerstung M., MacLean D., Timmer J., Fleck C., Lenhard M. (2007). Control of plant organ size by KLUH/CYP78A5-dependent intercellular signaling. Dev. Cell.

[36] Horváth B.M., Magyar Z., Zhang Y., Hamburger A.W., Bakó L., Visser R.G.F., Bachem C.W.B., Bögre L. (2006). EBP1 regulates organ size through cell growth and proliferation in plants. EMBO J..

[37] Li Y., Zheng L., Corke F., Smith C., Bevan M.W. (2008). Control of final seed and organ size by the DA1 gene family in Arabidopsis thaliana. Genes Dev..

[38] Peng Y., Chen L., Lu Y., Wu Y., Dumenil J., Zhu Z., Bevan M.W., Li Y. (2015). The ubiquitin receptors DA1, DAR1, and DAR2 redundantly regulate endoreduplication by modulating the stability of TCP14/15 in Arabidopsis. Plant Cell.

[39] Landoni M., Cassani E., Pilu R. (2007). Arabidopsis thaliana plants overexpressing Ramosa1 maize gene show an increase in organ size due to cell expansion. Sex. Plant Reprod..

[40] Li X., Liu W., Zhuang L., Zhu Y., Wang F., Chen T., Yang J., Ambrose M., Hu Z., Weller J.L. (2019). BIGGER ORGANS and ELEPHANT EAR-LIKE LEAF1 control organ size and floral organ internal asymmetry in pea. J. Exp. Bot..

[41] Roberts W.R., Roalson E.H. (2017). Comparative transcriptome analyses of flower development in four species of Achimenes (Gesneriaceae). BMC Genom..

[42] Almeida A.M.R., Piñeyro-Nelson A., Yockteng R.B., Specht C.D. (2018). Comparative analysis of whole flower transcriptomes in the Zingiberales. PeerJ.

[43] He W., Chen Y., Gao M., Zhao Y., Xu Z., Cao P., Zhang Q., Jiao Y., Li H., Wu L. (2018). Transcriptome Analysis of Litsea cubeba Floral Buds Reveals the Role of Hormones and Transcription Factors in the Differentiation Process. G3 Genes Genomes Genet..

[44] Wang S., Li Z., Jin W., Fang Y., Yang Q., Xiang J. (2018). Transcriptome analysis and identification of genes associated with flower development in Rhododendron pulchrum Sweet (Ericaceae). Gene.

[45] Yue J., Zhu C., Zhou Y., Niu X., Miao M., Tang X., Chen F., Zhao W., Liu Y. (2018). Transcriptome analysis of differentially expressed unigenes involved in flavonoid biosynthesis during flower development of Chrysanthemum morifolium “Chuju”. Sci. Rep..

[46] Liu K., Feng S., Pan Y., Zhong J., Chen Y., Yuan C., Li H. (2016). Transcriptome Analysis and Identification of Genes Associated with Floral Transition and Flower Development in Sugar Apple (Annona squamosa L.). Front. Plant Sci..

[47] Wang J., Wang H., Ding L., Song A., Shen F., Jiang J., Chen S., Chen F. (2017). Transcriptomic and hormone analyses reveal mechanisms underlying petal elongation in Chrysanthemum morifolium “Jinba”. Plant Mol. Biol..

[48] Cohen J.I. (2016). De novo Sequencing and Comparative Transcriptomics of Floral Development of the Distylous Species Lithospermum multiflorum. Front. Plant Sci..

[49] Liqin G., Jianguo Z., Xiaoxia L., Guodong R. (2019). Polyploidy-related differential gene expression between diploid and synthesized allotriploid and allotetraploid hybrids of Populus. Mol. Breed..

[50] Landis J.B., Soltis D.E., Soltis P.S. (2017). Comparative transcriptomic analysis of the evolution and development of flower size in Saltugilia (Polemoniaceae). BMC Genom..

[51] Leebens-Mack J.H., Barker M.S., Carpenter E.J., Deyholos M.K., Gitzendanner M.A., Graham S.W., Grosse I., Li Z., Melkonian M., Mirarab S. (2019). One thousand plant transcriptomes and the phylogenomics of green plants. Nature.

[52] Landis J.B., Soltis D.E., Li Z., Marx H.E., Barker M.S., Tank D.C., Soltis P.S. (2018). Impact of whole-genome duplication events on diversification rates in angiosperms. Am. J. Bot..

[53] Schneider H., Liu H.-M., Chang Y.-F., Ohlsen D., Perrie L.R., Shepherd L., Kessler M., Karger D.N., Hennequin S., Marquardt J. (2017). Neo- and Paleopolyploidy contribute to the species diversity of Asplenium the most species-rich genus of ferns: Polyploidy in Asplenium. J. Syst. Evol..

[54] Zhang R., Wang F.-G., Zhang J., Shang H., Liu L., Wang H., Zhao G.-H., Shen H., Yan Y.-H. (2019). Dating Whole Genome Duplication in Ceratopteris thalictroides and Potential Adaptive Values of Retained Gene Duplicates. Int. J. Mol. Sci..

[55] Han T.-S., Zheng Q.-J., Onstein R.E., Rojas-Andrés B.M., Hauenschild F., Muellner-Riehl A.N., Xing Y.-W. (2019). Polyploidy promotes species diversification of Allium through ecological shifts. New Phytol..

[56] Levin D.A. (2019). Plant speciation in the age of climate change. Ann. Bot..

[57] Bennett M.D. (1971). The duration of meiosis. Proc. R. Soc. Lond. Ser. B Biol. Sci..

[58] Beaulieu J.M., Leitch I.J., Patel S., Pendharkar A., Knight C.A. (2008). Genome size is a strong predictor of cell size and stomatal density in angiosperms. New Phytol..

[59] Snodgrass S.J., Jareczek J., Wendel J.F. (2017). An examination of nucleotypic effects in diploid and polyploid cotton. AoB Plants.

[60] Doyle J.J., Coate J.E. (2019). Polyploidy, the Nucleotype, and Novelty: The Impact of Genome Doubling on the Biology of the Cell. Int. J. Plant Sci..

[61] Roddy A.B., Théroux-Rancourt G., Abbo T., Benedetti J.W., Brodersen C.R., Castro M., Castro S., Gilbride A.B., Jensen B., Jiang G.-F. (2020). The Scaling of Genome Size and Cell Size Limits Maximum Rates of Photosynthesis with Implications for Ecological Strategies. Int. J. Plant Sci..

[62] Katagiri Y., Hasegawa J., Fujikura U., Hoshino R., Matsunaga S., Tsukaya H. (2016). The coordination of ploidy and cell size differs between cell layers in leaves. Development.

[63] Coate J.E., Luciano A.K., Seralathan V., Minchew K.J., Owens T.G., Doyle J.J. (2012). Anatomical, biochemical, and photosynthetic responses to recent allopolyploidy in Glycine dolichocarpa (Fabaceae). Am. J. Bot..

[64] Mishra M.K. (1997). Stomatal Characteristics at Different Ploidy Levels in *Coffea* L. Ann. Bot..

[65] Wong C., Murray B.G. (2012). Variable changes in genome size associated with different polyploid events in Plantago (Plantaginaceae). J. Hered..

[66] Kondorosi E., Roudier F., Gendreau E. (2000). Plant cell-size control: Growing by ploidy?. Curr. Opin. Plant Biol..

[67] Kudo N., Kimura Y. (2002). Nuclear DNA endoreduplication during petal development in cabbage: Relationship between ploidy levels and cell size. J. Exp. Bot..

[68] Sugimoto-Shirasu K., Roberts K. (2003). “Big it up”: Endoreduplication and cell-size control in plants. Curr. Opin. Plant Biol..

[69] Cheniclet C., Rong W.Y., Causse M., Frangne N., Bolling L., Carde J.-P., Renaudin J.-P. (2005). Cell Expansion and Endoreduplication Show a Large Genetic Variability in Pericarp and Contribute Strongly to Tomato Fruit Growth. Plant Physiol..

[70] Orr-Weaver T.L. (2015). When bigger is better: The role of polyploidy in organogenesis. Trends Genet..

[71] Simonin K.A., Roddy A.B. (2018). Genome downsizing, physiological novelty, and the global dominance of flowering plants. PLoS Biol..

[72] Brochmann C., Brysting A.K., Alsos I.G., Borgen L., Grundt H.H., Scheen A.-C., Elven R. (2004). Polyploidy in arctic plants. Biol. J. Linn. Soc. Lond..

[73] Leitch A.R., Leitch I.J. (2008). Genomic plasticity and the diversity of polyploid plants. Science.

[74] Doyle J.J., Flagel L.E., Paterson A.H., Rapp R.A., Soltis D.E., Soltis P.S., Wendel J.F. (2008). Evolutionary genetics of genome merger and doubling in plants. Annu. Rev. Genet..

[75] Te Beest M., Le Roux J.J., Richardson D.M., Brysting A.K., Suda J., Kubesová M., Pysek P. (2012). The more the better? The role of polyploidy in facilitating plant invasions. Ann. Bot..

[76] Edger P.P., Heidel-Fischer H.M., Bekaert M., Rota J., Glöckner G., Platts A.E., Heckel D.G., Der J.P., Wafula E.K., Tang M. (2015). The butterfly plant arms-race escalated by gene and genome duplications. Proc. Natl. Acad. Sci. USA.

[77] Visger C.J., Germain-Aubrey C.C., Patel M., Sessa E.B., Soltis P.S., Soltis D.E. (2016). Niche divergence between diploid and autotetraploid Tolmiea. Am. J. Bot..

[78] Tate J.A., Symonds V.V., Doust A.N., Buggs R.J.A., Mavrodiev E., Majure L.C., Soltis P.S., Soltis D.E. (2009). Synthetic polyploids of Tragopogon miscellus and T. mirus (Asteraceae): 60 Years after Ownbey’s discovery. Am. J. Bot..

[79] Malek M.A., Ismail M.R., Rafii M.Y., Rahman M. (2012). Synthetic Brassica napus L.: Development and studies on morphological characters, yield attributes, and yield. Sci. World J..

[80] Zhang X., Liu T., Li X., Duan M., Wang J., Qiu Y., Wang H., Song J., Shen D. (2016). Interspecific hybridization, polyploidization, and backcross of Brassica oleracea var. alboglabra with B. rapa var. purpurea morphologically recapitulate the evolution of Brassica vegetables. Sci. Rep..

[81] Pavlíková Z., Paštová L., Münzbergová Z. (2017). Synthetic polyploids in Vicia cracca: Methodology, effects on plant performance and aneuploidy. Plant Syst. Evol..

[82] Wei N., Du Z., Liston A., Ashman T.-L. (2019). Genome duplication effects on functional traits and fitness are genetic context and species dependent: Studies of synthetic polyploid Fragaria. Am. J. Bot..

[83] Forrester N.J., Ashman T.-L. (2019). Autopolyploidy alters nodule-level interactions in the legume-rhizobium mutualism. Am. J. Bot..

[84] Chase M.W., Knapp S., Cox A.V., Clarkson J.J., Butsko Y., Joseph J., Savolainen V., Parokonny A.S. (2003). Molecular systematics, GISH and the origin of hybrid taxa in Nicotiana (Solanaceae). Ann. Bot..

[85] Clarkson J.J., Knapp S., Garcia V.F., Olmstead R.G., Leitch A.R., Chase M.W. (2004). Phylogenetic relationships in Nicotiana (Solanaceae) inferred from multiple plastid DNA regions. Mol. Phylogenet. Evol..

[86] Clarkson J.J., Kelly L.J., Leitch A.R., Knapp S., Chase M.W. (2010). Nuclear glutamine synthetase evolution in Nicotiana: Phylogenetics and the origins of allotetraploid and homoploid (diploid) hybrids. Mol. Phylogenet. Evol..

[87] Clarkson J.J., Dodsworth S., Chase M.W. (2017). Time-calibrated phylogenetic trees establish a lag between polyploidisation and diversification in Nicotiana (Solanaceae). Plant Syst. Evol..

[88] Kelly L.J., Leitch A.R., Clarkson J.J., Hunter R.B., Knapp S., Chase M.W. (2010). Intragenic recombination events and evidence for hybrid speciation in Nicotiana (Solanaceae). Mol. Biol. Evol..

[89] Kelly L.J., Leitch A.R., Clarkson J.J., Knapp S., Chase M.W. (2013). Reconstructing the complex evolutionary origin of wild allopolyploid tobaccos (Nicotiana section suaveolentes). Evolution.

[90] McCarthy E.W., Landis J.B., Kurti A., Lawhorn A.J., Chase M.W., Knapp S., Le Comber S.C., Leitch A.R., Litt A. (2019). Early consequences of allopolyploidy alter floral evolution in Nicotiana (Solanaceae). BMC Plant Biol..

[91] McCarthy E.W., Chase M.W., Knapp S., Litt A., Leitch A.R., Le Comber S.C. (2016). Transgressive phenotypes and generalist pollination in the floral evolution of Nicotiana polyploids. Nat. Plants.

[92] Zenil-Ferguson R., Burleigh J.G., Freyman W.A., Igić B., Mayrose I., Goldberg E.E. (2019). Interaction among ploidy, breeding system and lineage diversification. New Phytol..

[93] Fregonezi J.N., Turchetto C., Bonatto S.L., Freitas L.B. (2013). Biogeographical history and diversification of Petunia and Calibrachoa (Solanaceae) in the Neotropical Pampas grassland. Bot. J. Linn. Soc..

[94] Reck-Kortmann M., Silva-Arias G.A., Segatto A.L.A., Mäder G., Bonatto S.L., de Freitas L.B. (2014). Multilocus phylogeny reconstruction: New insights into the evolutionary history of the genus Petunia. Mol. Phylogenet. Evol..

[95] Teixeira M.C., Turchetto C., Maestri R., Freitas L.B. (2020). Morphological characterization of sympatric and allopatric populations of Petunia axillaris and P. exserta (Solanaceae). Bot. J. Linn. Soc..

[96] Edwards E.J., Chatelet D.S., Chen B.-C., Ong J.Y., Tagane S., Kanemitsu H., Tagawa K., Teramoto K., Park B., Chung K.-F. (2017). Convergence, Consilience, and the Evolution of Temperate Deciduous Forests. Am. Nat..

[97] Donoghue M.J., Edwards E.J. (2019). Model clades are vital for comparative biology, and ascertainment bias is not a problem in practice: A response to Beaulieu and O’Meara (2018). Am. J. Bot..

[98] Schneider C.A., Rasband W.S., Eliceiri K.W. (2012). NIH Image to ImageJ: 25 Years of image analysis. Nat. Methods.

[99] R Core team (2017). R: A Language and Environment for Statistical Computing.

[100] Kuznetsova A., Brockhoff P., Christensen R. (2017). lmerTest Package: Tests in Linear Mixed Effects Models. J. Stat. Softw. Artic..

[101] Lenth R. (2016). Least-Squares Means: The R Package lsmeans. J. Stat. Softw. Artic..

[102] Wickham H. (2016). Ggplot2: Elegant Graphics for Data Analysis.

[103] Zhong S., Joung J.-G., Zheng Y., Chen Y.-R., Liu B., Shao Y., Xiang J.Z., Fei Z., Giovannoni J.J. (2011). High-throughput illumina strand-specific RNA sequencing library preparation. Cold Spring Harb. Protoc..

[104] Martin M. (2011). Cutadapt removes adapter sequences from high-throughput sequencing reads. EMBnet. J..

[105] Wang J.R., Holt J., McMillan L., Jones C.D. (2018). FMLRC: Hybrid long read error correction using an FM-index. BMC Bioinform..

[106] Holt J., McMillan L. (2014). Merging of multi-string BWTs with applications. Bioinformatics.

[107] Zhang H., Jain C., Aluru S. (2019). A comprehensive evaluation of long read error correction methods. bioRxiv.

[108] Fu S., Wang A., Au K.F. (2019). A comparative evaluation of hybrid error correction methods for error-prone long reads. Genome Biol..

[109] Shen W., Le S., Li Y., Hu F. (2016). SeqKit: A Cross-Platform and Ultrafast Toolkit for FASTA/Q File Manipulation. PLoS ONE.

[110] Sierro N., Battey J.N.D., Ouadi S., Bovet L., Goepfert S., Bakaher N., Peitsch M.C., Ivanov N.V. (2013). Reference genomes and transcriptomes of Nicotiana sylvestris and Nicotiana tomentosiformis. Genome Biol..

[111] Edwards K.D., Fernandez-Pozo N., Drake-Stowe K., Humphry M., Evans A.D., Bombarely A., Allen F., Hurst R., White B., Kernodle S.P. (2017). A reference genome for Nicotiana tabacum enables map-based cloning of homeologous loci implicated in nitrogen utilization efficiency. BMC Genom..

[112] Simão F.A., Waterhouse R.M., Ioannidis P., Kriventseva E.V., Zdobnov E.M. (2015). BUSCO: Assessing genome assembly and annotation completeness with single-copy orthologs. Bioinformatics.

[113] Nurk S., Bankevich A., Antipov D., Gurevich A., Korobeynikov A., Lapidus A., Prjibelsky A., Pyshkin A., Sirotkin A., Sirotkin Y. (2013). Assembling Genomes and Mini-metagenomes from Highly Chimeric Reads. Proceedings of the Research in Computational Molecular Biology.

[114] Grabherr M.G., Haas B.J., Yassour M., Levin J.Z., Thompson D.A., Amit I., Adiconis X., Fan L., Raychowdhury R., Zeng Q. (2011). Full-length transcriptome assembly from RNA-Seq data without a reference genome. Nat. Biotechnol..

[115] Haas B.J., Papanicolaou A., Yassour M., Grabherr M., Blood P.D., Bowden J., Couger M.B., Eccles D., Li B., Lieber M. (2013). De novo transcript sequence reconstruction from RNA-seq using the Trinity platform for reference generation and analysis. Nat. Protoc..

[116] Robertson G., Schein J., Chiu R., Corbett R., Field M., Jackman S.D., Mungall K., Lee S., Okada H.M., Qian J.Q. (2010). De novo assembly and analysis of RNA-seq data. Nat. Methods.

[117] Tan G., Polychronopoulos D., Lenhard B. (2019). CNEr: A toolkit for exploring extreme noncoding conservation. PLoS Comput. Biol..

[118] Bryant D.M., Johnson K., DiTommaso T., Tickle T., Couger M.B., Payzin-Dogru D., Lee T.J., Leigh N.D., Kuo T.-H., Davis F.G. (2017). A Tissue-Mapped Axolotl De Novo Transcriptome Enables Identification of Limb Regeneration Factors. Cell Rep..

[119] Li B., Dewey C.N. (2011). RSEM: Accurate transcript quantification from RNA-Seq data with or without a reference genome. BMC Bioinform..

[120] Langmead B., Trapnell C., Pop M., Salzberg S.L. (2009). Ultrafast and memory-efficient alignment of short DNA sequences to the human genome. Genome Biol..

[121] Camacho C., Coulouris G., Avagyan V., Ma N., Papadopoulos J., Bealer K., Madden T.L. (2009). BLAST+: Architecture and applications. BMC Bioinform..

[122] Xu L., Dong Z., Fang L., Luo Y., Wei Z., Guo H., Zhang G., Gu Y.Q., Coleman-Derr D., Xia Q. (2019). OrthoVenn2: A web server for whole-genome comparison and annotation of orthologous clusters across multiple species. Nucleic Acids Res..

[123] Ritchie M.E., Phipson B., Wu D., Hu Y., Law C.W., Shi W., Smyth G.K. (2015). limma powers differential expression analyses for RNA-sequencing and microarray studies. Nucleic Acids Res..

[124] Li H., Handsaker B., Wysoker A., Fennell T., Ruan J., Homer N., Marth G., Abecasis G., Durbin R. (2009). 1000 Genome Project Data Processing Subgroup The Sequence Alignment/Map format and SAMtools. Bioinformatics.

[125] Robinson J.T., Thorvaldsdóttir H., Winckler W., Guttman M., Lander E.S., Getz G., Mesirov J.P. (2011). Integrative genomics viewer. Nat. Biotechnol..

[126] Thorvaldsdóttir H., Robinson J.T., Mesirov J.P. (2013). Integrative Genomics Viewer (IGV): High-performance genomics data visualization and exploration. Brief. Bioinform..

[127] Robinson J.T., Thorvaldsdóttir H., Wenger A.M., Zehir A., Mesirov J.P. (2017). Variant Review with the Integrative Genomics Viewer. Cancer Res..

[128] Landis J.B., O’Toole R.D., Ventura K.L., Gitzendanner M.A., Oppenheimer D.G., Soltis D.E., Soltis P.S. (2016). The phenotypic and genetic underpinnings of flower size in Polemoniaceae. Front. Plant Sci..

[129] Zhurinsky J., Leonhard K., Watt S., Marguerat S., Bähler J., Nurse P. (2010). A coordinated global control over cellular transcription. Curr. Biol..

[130] Marguerat S., Bähler J. (2012). Coordinating genome expression with cell size. Trends Genet..

[131] Ietswaart R., Rosa S., Wu Z., Dean C., Howard M. (2017). Cell-Size-Dependent Transcription of FLC and Its Antisense Long Non-coding RNA COOLAIR Explain Cell-to-Cell Expression Variation. Cell Syst.

[132] Wu C.-Y., Rolfe P.A., Gifford D.K., Fink G.R. (2010). Control of transcription by cell size. PLoS Biol..

[133] Padovan-Merhar O., Nair G.P., Biaesch A.G., Mayer A., Scarfone S., Foley S.W., Wu A.R., Churchman L.S., Singh A., Raj A. (2015). Single mammalian cells compensate for differences in cellular volume and DNA copy number through independent global transcriptional mechanisms. Mol. Cell.

[134] Schoenfelder K.P., Fox D.T. (2015). The expanding implications of polyploidy. J. Cell Biol..

[135] Jaakola L., Määttä K., Pirttilä A.M., Törrönen R., Kärenlampi S., Hohtola A. (2002). Expression of genes involved in anthocyanin biosynthesis in relation to anthocyanin, proanthocyanidin, and flavonol levels during bilberry fruit development. Plant Physiol..

[136] Wilhelmsson P.K.I., Chandler J.O., Fernandez-Pozo N., Graeber K., Ullrich K.K., Arshad W., Khan S., Hofberger J.A., Buchta K., Edger P.P. (2019). Usability of reference-free transcriptome assemblies for detection of differential expression: A case study on Aethionema arabicum dimorphic seeds. BMC Genom..

[137] Holding M.L., Margres M.J., Mason A.J., Parkinson C.L., Rokyta D.R. (2018). Evaluating the Performance of De Novo Assembly Methods for Venom-Gland Transcriptomics. Toxins.

[138] Hsieh P.-H., Oyang Y.-J., Chen C.-Y. (2019). Effect of de novo transcriptome assembly on transcript quantification. Sci. Rep..

[139] McCarthy E.W., Landis J.B., Kurti A., Lawhorn A.J., Litt A., Ivanov N.V., Sierro N., Peitsch M.C. (2020). The Genetic Basis of Flower Color Differences in Nicotiana tabacum. The Tobacco Plant Genome.

[140] Francis D., Davies M.S., Barlow P.W. (2008). A strong nucleotypic effect on the cell cycle regardless of ploidy level. Ann. Bot..

[141] Robinson D.O., Coate J.E., Singh A., Hong L., Bush M., Doyle J.J., Roeder A.H.K. (2018). Ploidy and Size at Multiple Scales in the Arabidopsis Sepal. Plant Cell.

[142] Miller M., Zhang C., Chen Z.J. (2012). Ploidy and Hybridity Effects on Growth Vigor and Gene Expression in Arabidopsis thaliana Hybrids and Their Parents. G3 Genes Genomes Genet..

[143] Shao J., Chen C., Deng X. (2003). In vitro induction of tetraploid in pomegranate (*Punica granatum*). Plant Cell Tissue Organ Cult..

[144] Zhang Z., Dai H., Xiao M., Liu X. (2008). In vitro induction of tetraploids in *Phlox subulata* L. Euphytica.

[145] Gantait S., Mandal N., Bhattacharyya S., Das P.K. (2011). Induction and identification of tetraploids using in vitro colchicine treatment of *Gerbera jamesonii* Bolus cv. Sciella. Plant Cell Tissue Organ Cult..

[146] Vamosi J.C., Goring S.J., Kennedy B.F., Mayberry R.J., Moray C.M., Neame L.A., Tunbridge N.D., Elle E. (2007). Pollination, floral display, and the ecological correlates of polyploidy. Funct. Ecosyst. Communities.

[147] Gaeta R.T., Pires J.C., Iniguez-Luy F., Leon E., Osborn T.C. (2007). Genomic Changes in Resynthesized Brassica napus and Their Effect on Gene Expression and Phenotype. Plant Cell.

[148] Leitch I.J., Hanson L., Lim K.Y., Kovarik A., Chase M.W., Clarkson J.J., Leitch A.R. (2008). The ups and downs of genome size evolution in polyploid species of Nicotiana (Solanaceae). Ann. Bot..

[149] Münzbergová Z. (2017). Colchicine application significantly affects plant performance in the second generation of synthetic polyploids and its effects vary between populations. Ann. Bot..

[150] McCarthy E.W., Arnold S.E.J., Chittka L., Le Comber S.C., Verity R., Dodsworth S., Knapp S., Kelly L.J., Chase M.W., Baldwin I.T. (2015). The effect of polyploidy and hybridization on the evolution of floral colour in Nicotiana (Solanaceae). Ann. Bot..

