# Supplementary material for: Differential Gene Expression with an Emphasis on Floral Organ Size Differences in Natural and Synthetic Polyploids of Nicotiana tabacum (Solanaceae)
Source: Genes (Basel). 2020 Sep 19;11(9):1097. doi: 10.3390/genes11091097 (PMC7563459; doi:10.3390/genes11091097)
Supplement: Supplementary file 1 [file genes-11-01097-s001.zip › Supplemental Figure S5.pdf]

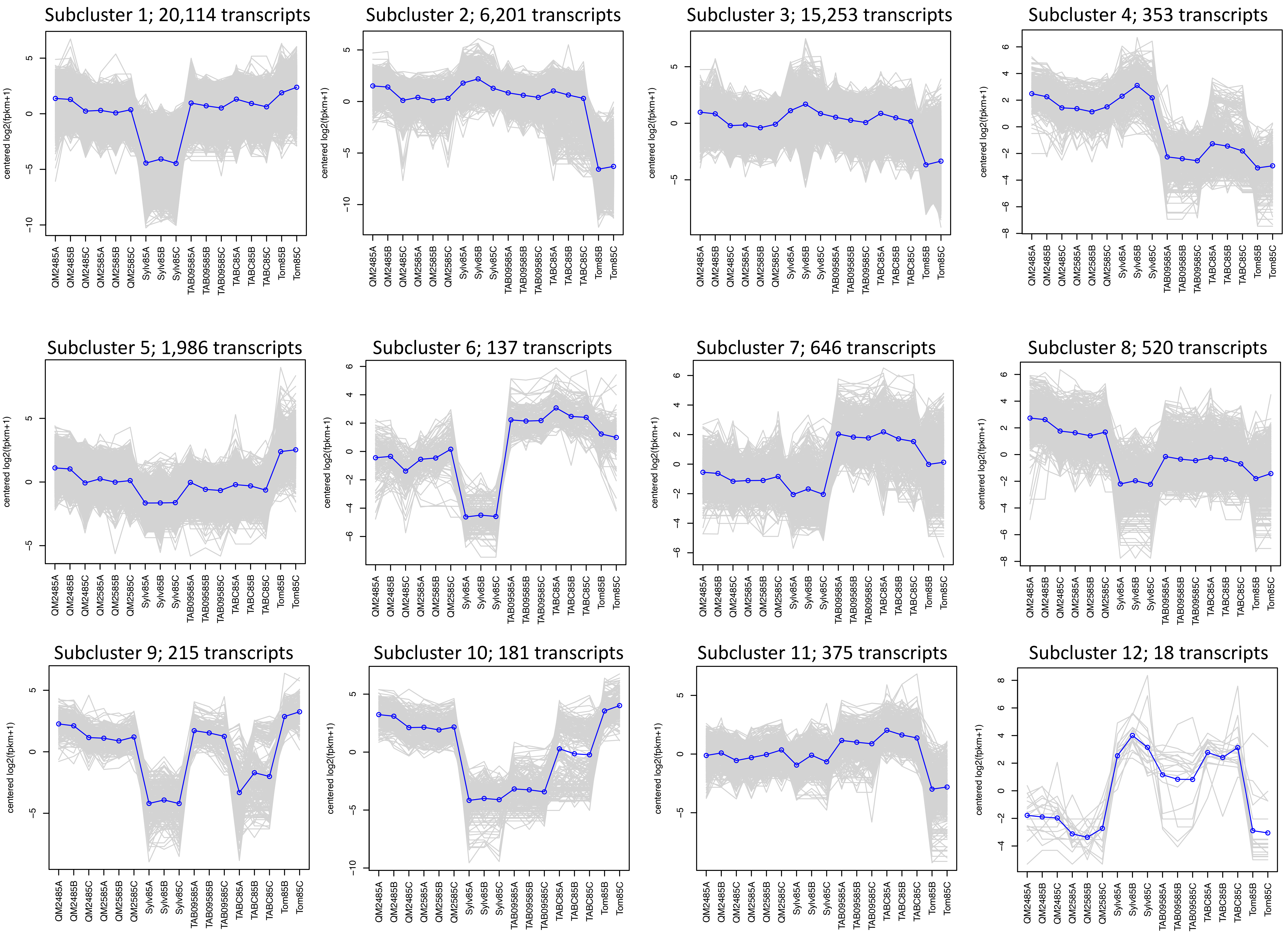
Subcluster 1; 20,114 transcripts
Subcluster 2; 6,201 transcripts
Subcluster 3; 15,253 transcripts
Subcluster 4; 353 transcripts
Subcluster 5; 1,986 transcripts
Subcluster 6; 137 transcripts
Subcluster 7; 646 transcripts
Subcluster 8; 520 transcripts
Subcluster 9; 215 transcripts
Subcluster 10; 181 transcripts
Subcluster 11; 375 transcripts
Subcluster 12; 18 transcripts
centered log2(fpkm+1)
QM2485A
QM2485B
QM2485C
QM2585A
QM2585B
QM2585C
Sylv85A
Sylv85B
Sylv85C
TAB09585A
TAB09585B
TAB09585C
TABC85A
TABC85B
TABC85C
Tom85B
Tom85C
