# Supplementary material for: Differential Gene Expression with an Emphasis on Floral Organ Size Differences in Natural and Synthetic Polyploids of Nicotiana tabacum (Solanaceae)
Source: Genes (Basel). 2020 Sep 19;11(9):1097. doi: 10.3390/genes11091097 (PMC7563459; doi:10.3390/genes11091097)
Supplement: Supplementary file 1 [file genes-11-01097-s001.zip › Supplemental Figure S6.pdf]

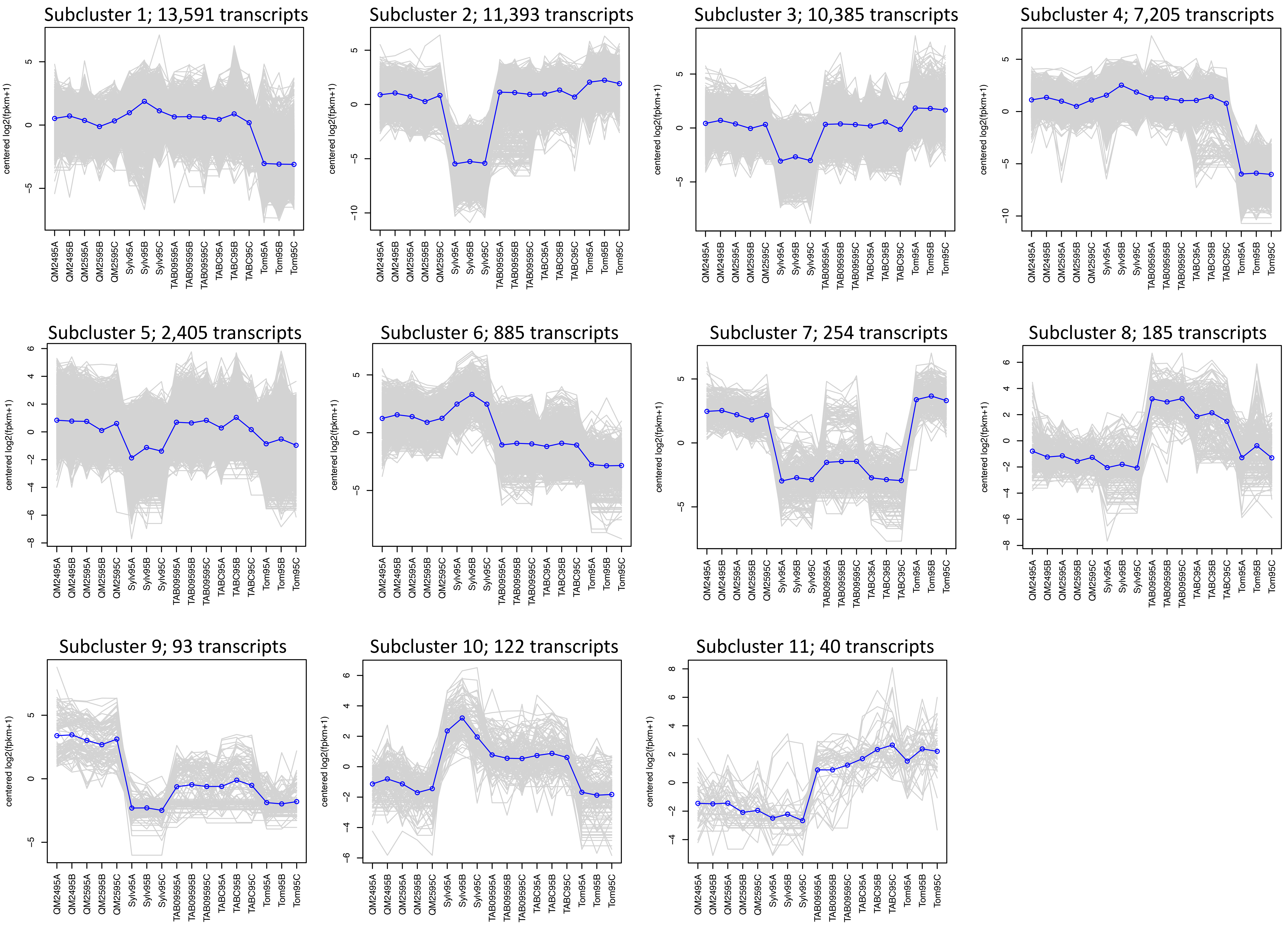
Subcluster 1; 13,591 transcripts
Subcluster 2; 11,393 transcripts
Subcluster 3; 10,385 transcripts
Subcluster 4; 7,205 transcripts
Subcluster 5; 2,405 transcripts
Subcluster 6; 885 transcripts
Subcluster 7; 254 transcripts
Subcluster 8; 185 transcripts
Subcluster 9; 93 transcripts
Subcluster 10; 122 transcripts
Subcluster 11; 40 transcripts
centered log2(fpkm+1)
QM2495A
QM2495B
QM2595A
QM2595B
QM2595C
Sylv95A
Sylv95B
Sylv95C
TAB09595A
TAB09595B
TAB09595C
TABC95A
TABC95B
TABC95C
Tom95A
Tom95B
Tom95C
