# Supplementary material for: Differential Gene Expression with an Emphasis on Floral Organ Size Differences in Natural and Synthetic Polyploids of Nicotiana tabacum (Solanaceae)
Source: Genes (Basel). 2020 Sep 19;11(9):1097. doi: 10.3390/genes11091097 (PMC7563459; doi:10.3390/genes11091097)
Supplement: Supplementary file 1 [file genes-11-01097-s001.zip › Supplemental Table S2.docx]

Table S2. Pairwise comparisons of Ploidy categories (diploid, natural polyploid, and synthetic polyploid) at minimum, 1^st^ quartile, median, 3^rd^ quartile, and maximum values of Width for Corolla tube circumference~Width + Ploidy + Width:Ploidy + Cell number model.

| **Minimum: Width=13.1** | **Estimate** | **SE** | **df** | **t ratio** | **p value** |
| --- | --- | --- | --- | --- | --- |
| diploid-natural polyploid | -3.36 | 1.27 | 21 | -2.64 | 0.04 |
| diploid-synthetic polyploid | -3.38 | 1.68 | 21 | -2.12 | 0.13 |
| natural polyploid-synthetic polyploid | -0.02 | 2.08 | 21 | -0.008 | 1.00 |
| **1^st^ quartile: Width=25.9** | **Estimate** | **SE** | **df** | **t ratio** | **p value** |
| diploid-natural polyploid | 0.03 | 0.36 | 21 | 0.10 | 1.00 |
| diploid-synthetic polyploid | 0.14 | 0.69 | 21 | 0.21 | 0.98 |
| natural polyploid-synthetic polyploid | 0.11 | 0.66 | 21 | 0.16 | 0.99 |
| **Median: Width=29.0** | **Estimate** | **SE** | **df** | **t ratio** | **p value** |
| diploid-natural polyploid | 0.87 | 0.45 | 21 | 1.94 | 0.15 |
| diploid-synthetic polyploid | 1.01 | 0.54 | 21 | 1.88 | 0.17 |
| natural polyploid-synthetic polyploid | 0.14 | 0.45 | 21 | 0.31 | 0.95 |
| **3^rd^ quartile: Width=31.8** | **Estimate** | **SE** | **df** | **t ratio** | **p value** |
| diploid-natural polyploid | 1.60 | 0.64 | 21 | 2.51 | 0.05 |
| diploid-synthetic polyploid | 1.77 | 0.50 | 21 | 3.51 | 0.006 |
| natural polyploid-synthetic polyploid | 0.16 | 0.47 | 21 | 0.35 | 0.94 |
| **Maximum: Width=37.2** | **Estimate** | **SE** | **df** | **t ratio** | **p value** |
| diploid-natural polyploid | 3.06 | 1.10 | 21 | 2.77 | 0.03 |
| diploid-synthetic polyploid | 3.28 | 0.73 | 21 | 4.48 | 6x10^-4^ |
| natural polyploid-synthetic polyploid | 0.22 | 0.96 | 21 | 0.23 | 0.97 |
