# Supplementary figures and images for: Differential Gene Expression with an Emphasis on Floral Organ Size Differences in Natural and Synthetic Polyploids of Nicotiana tabacum (Solanaceae)
Source: Genes (Basel). 2020 Sep 19;11(9):1097. doi: 10.3390/genes11091097 (PMC7563459; doi:10.3390/genes11091097)

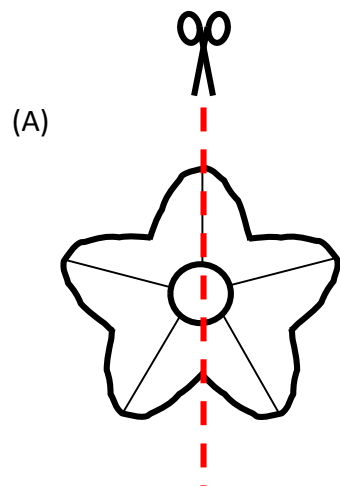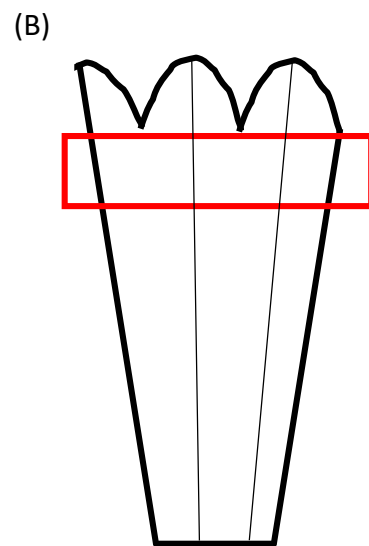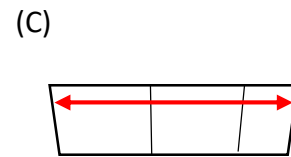

Supplement: Supplementary file 1 [file genes-11-01097-s001.zip › Supplemental Figure S1.pdf]

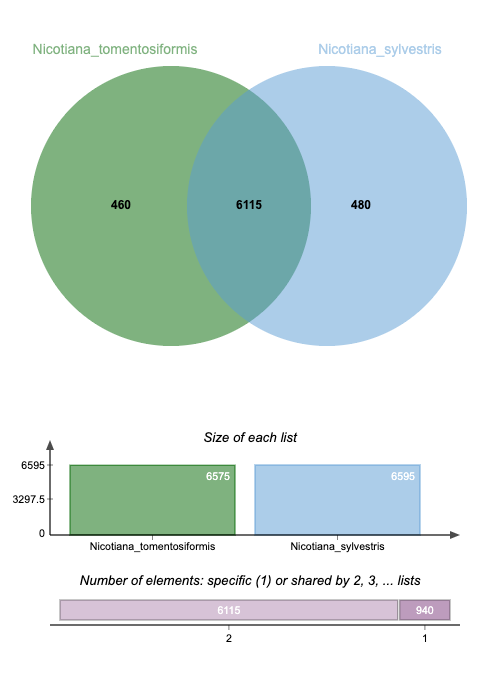

Supplement: Supplementary file 1 [file genes-11-01097-s001.zip › Supplemental Figure S2.png]

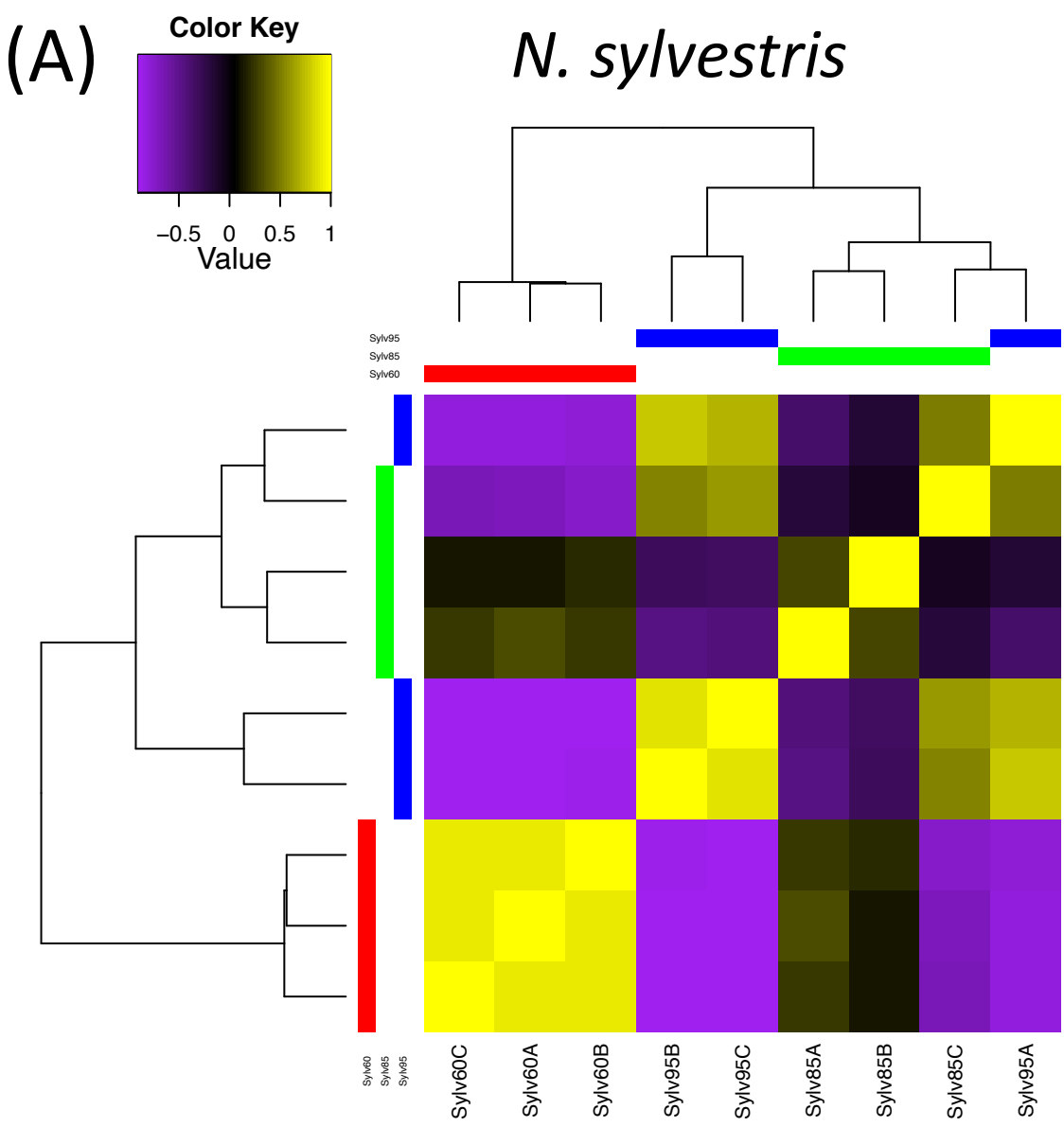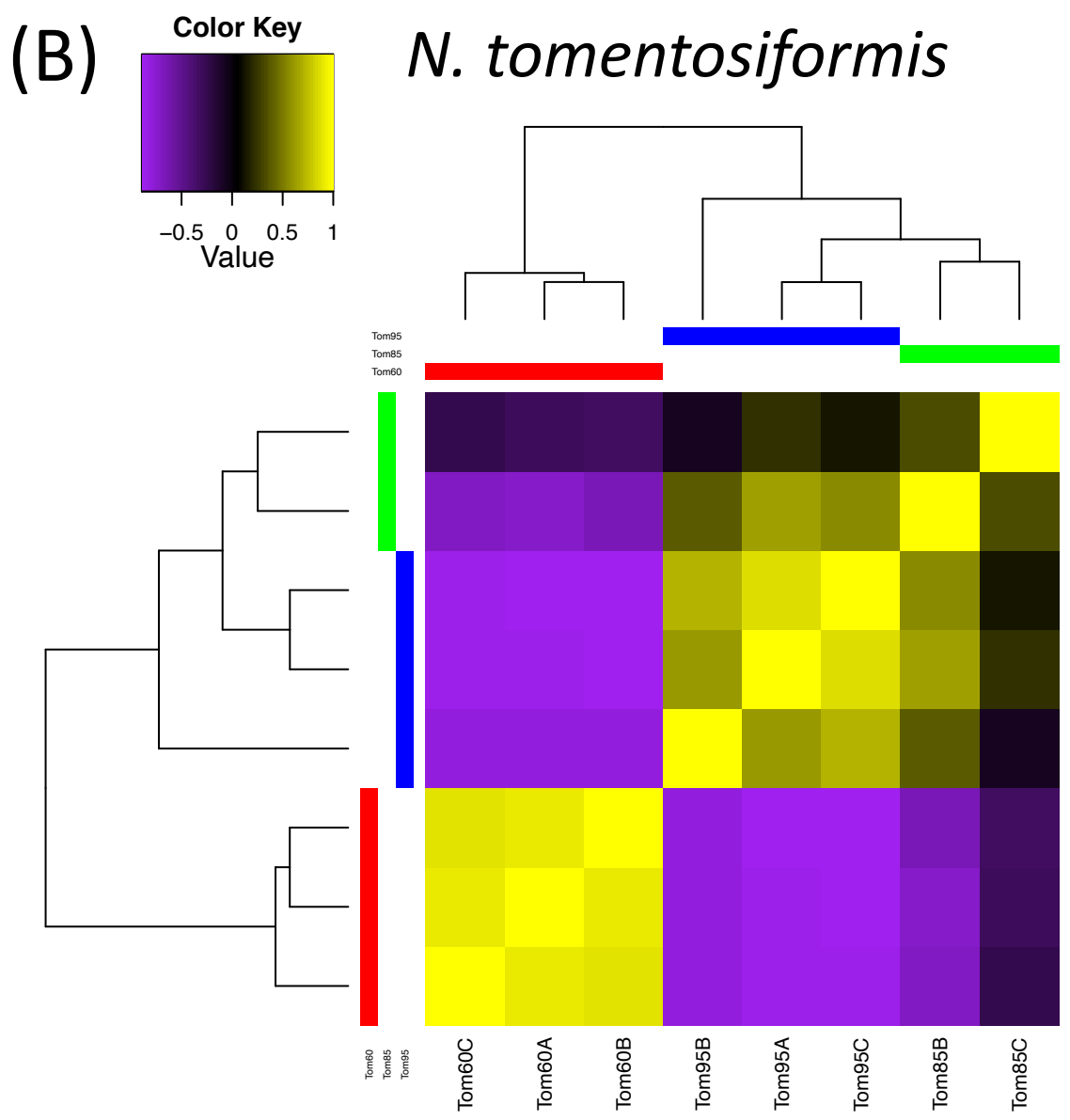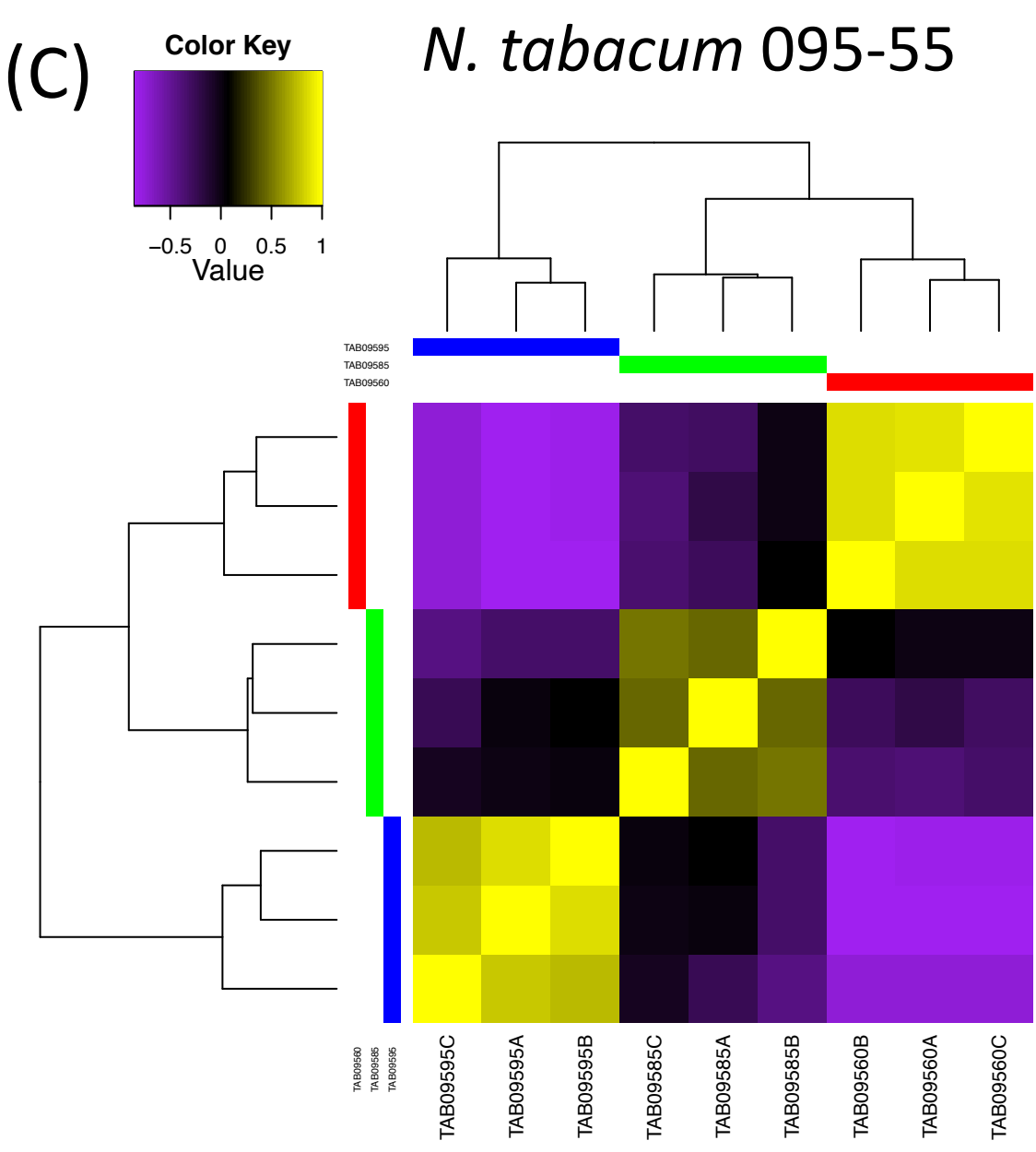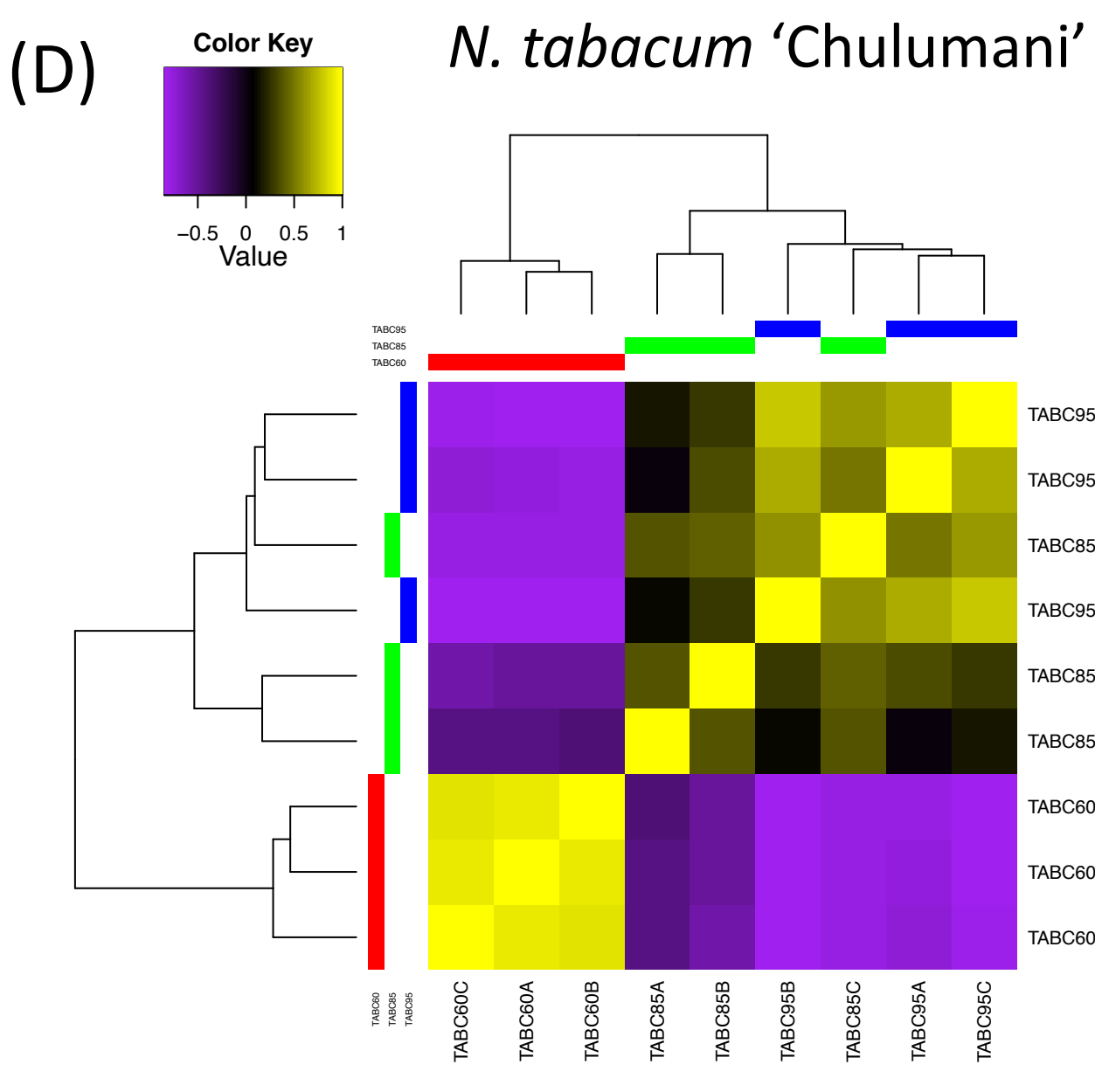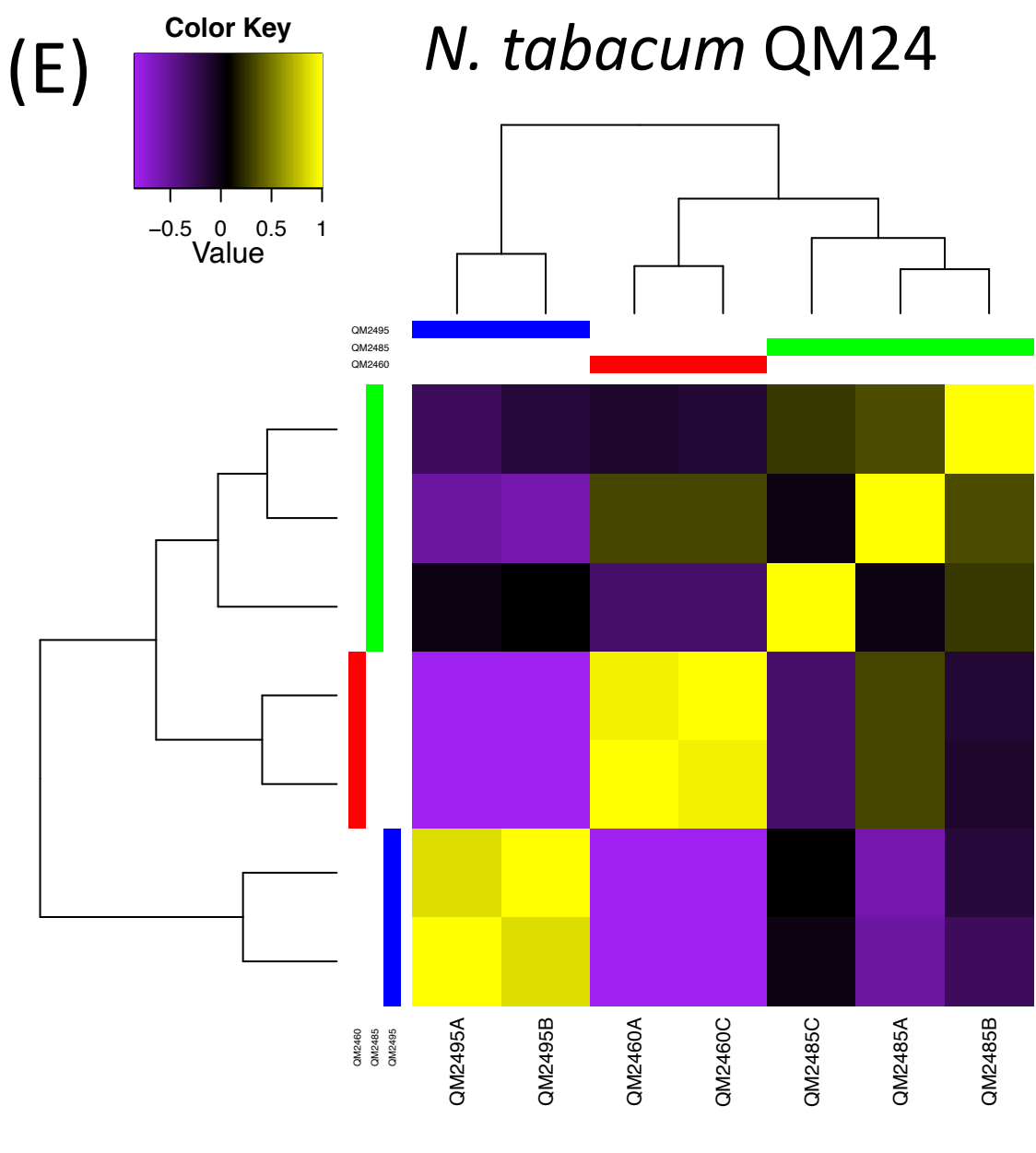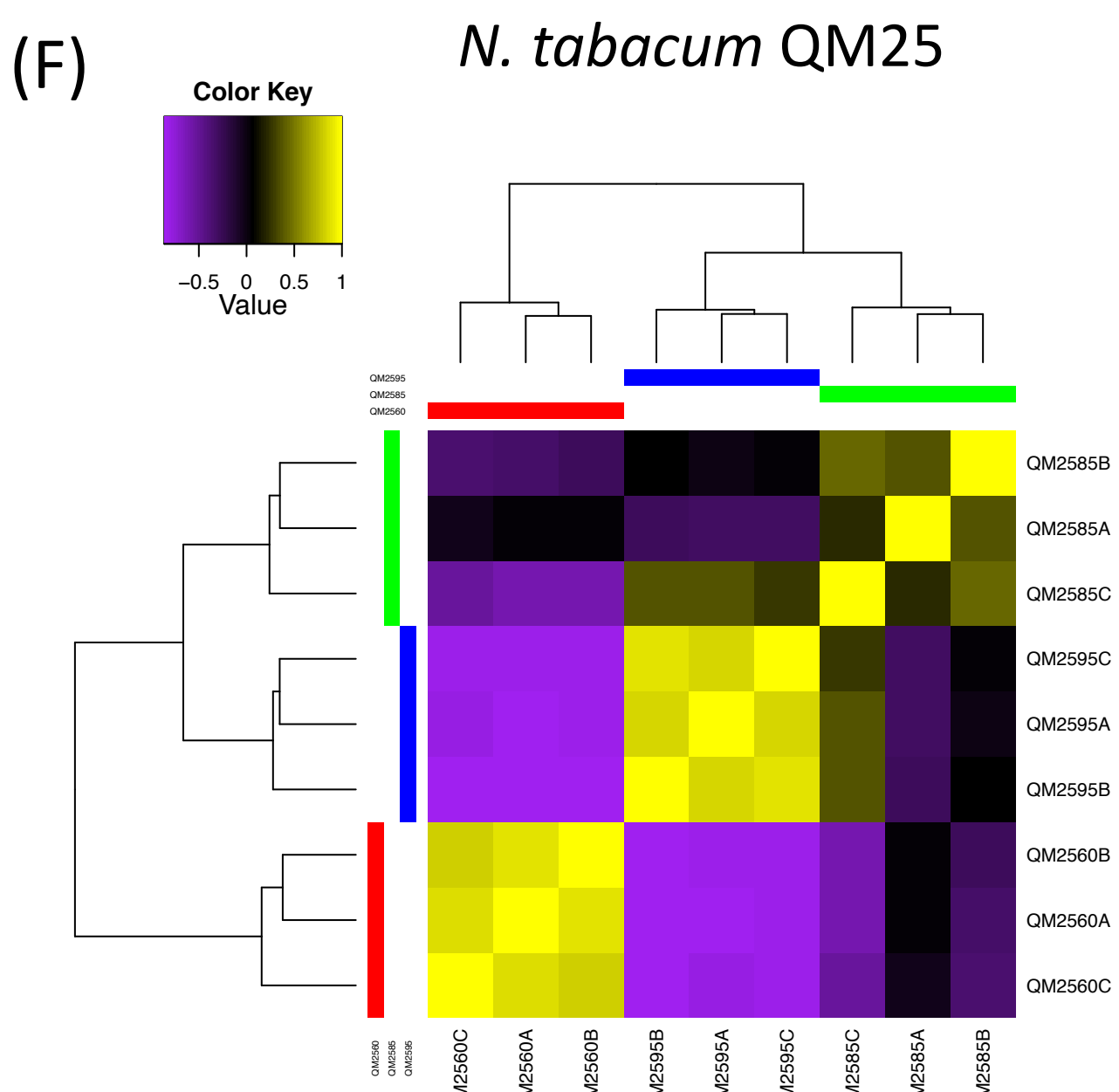

Supplement: Supplementary file 1 [file genes-11-01097-s001.zip › Supplemental Figure S3.pdf]

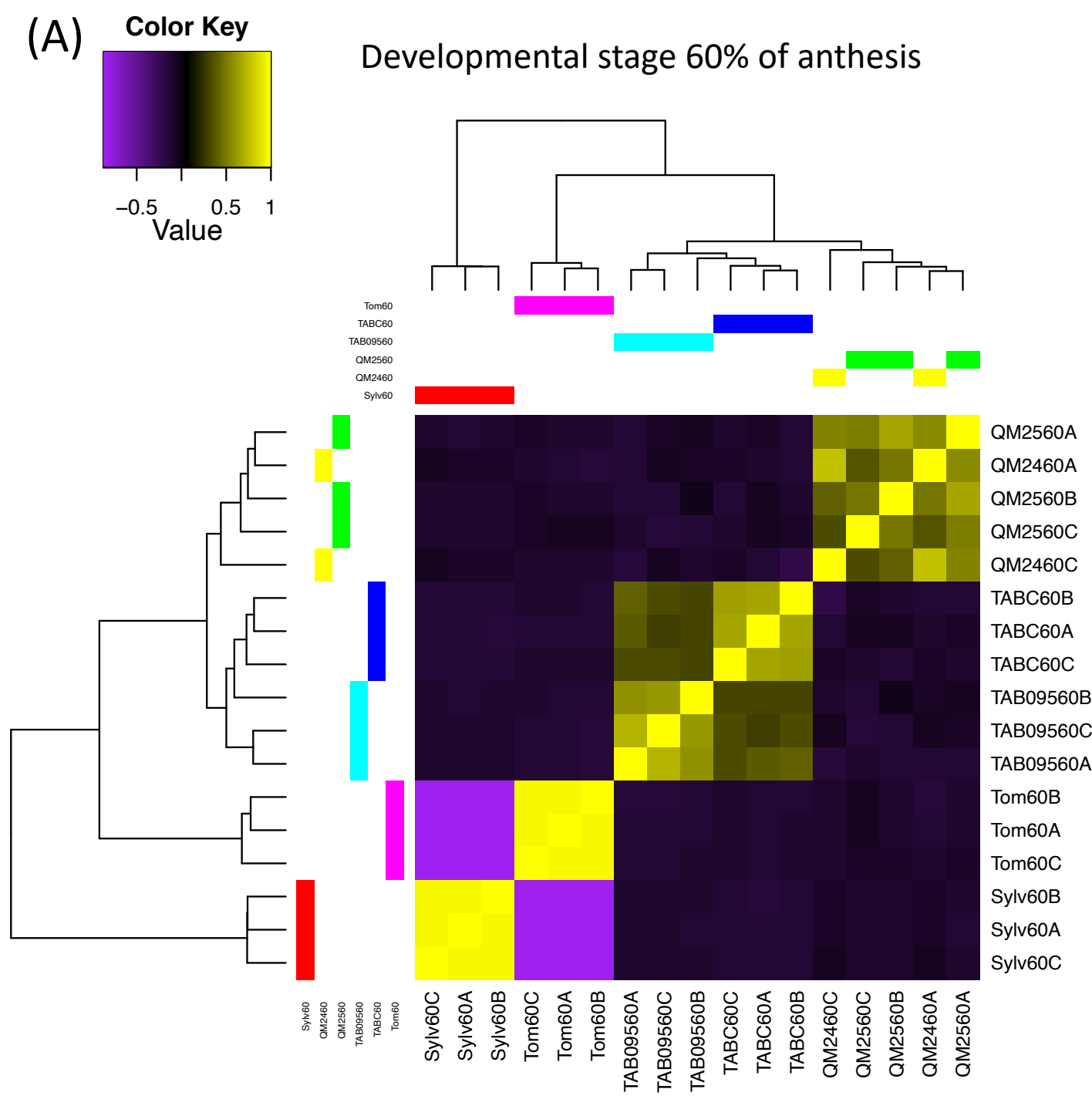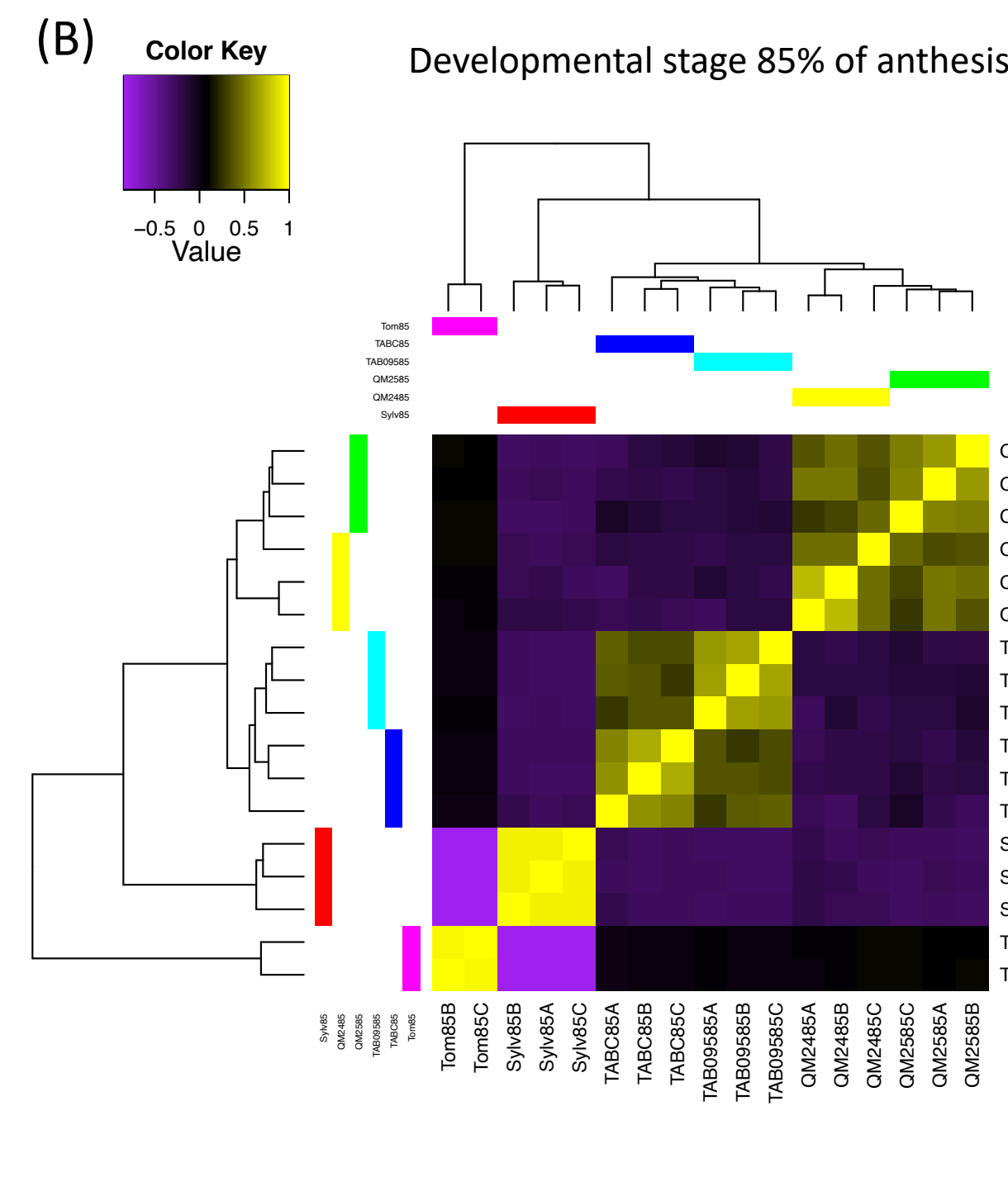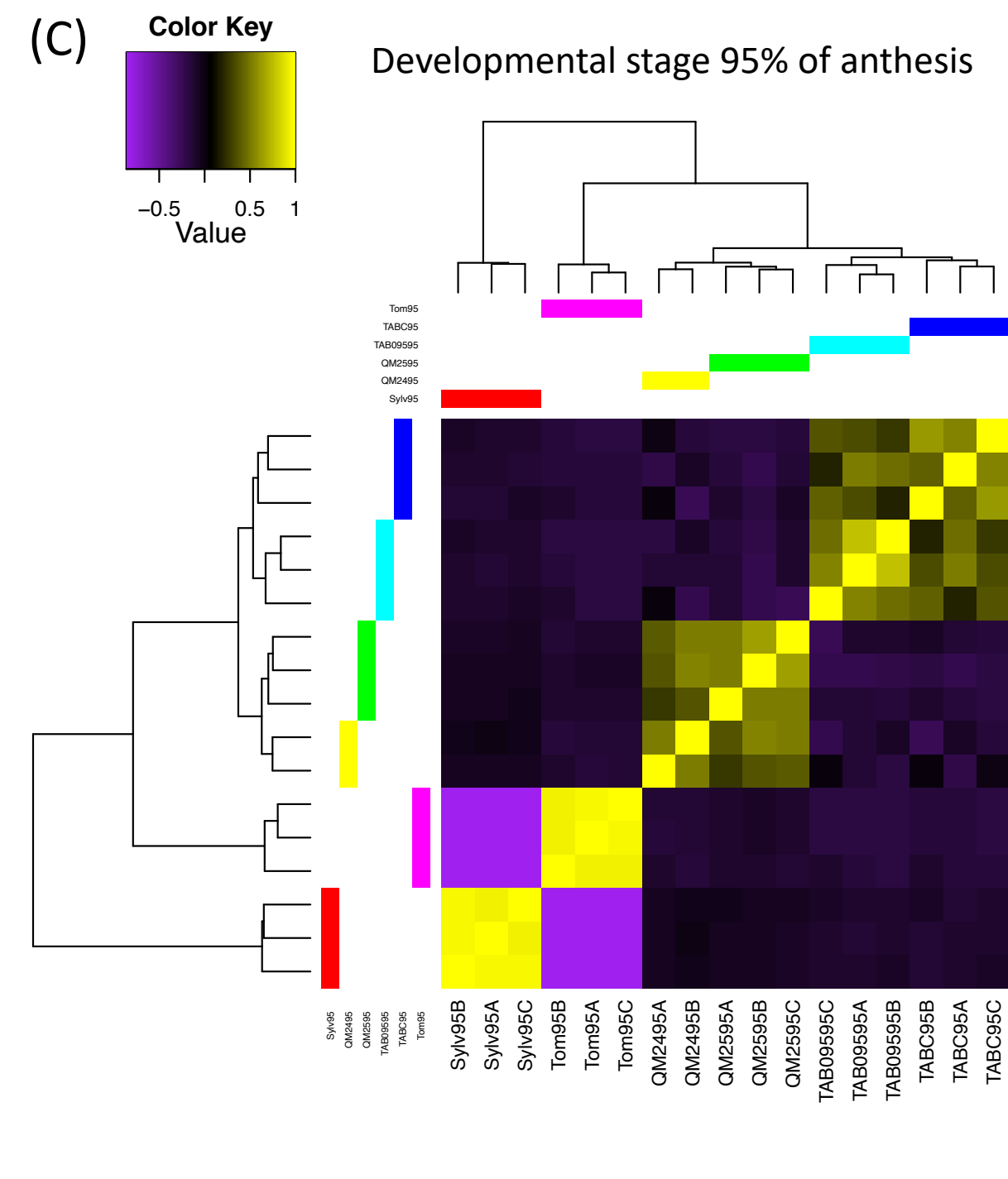

Supplement: Supplementary file 1 [file genes-11-01097-s001.zip › Supplemental Figure S4.pdf]

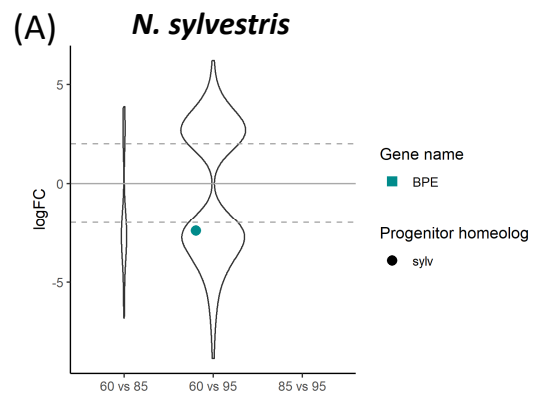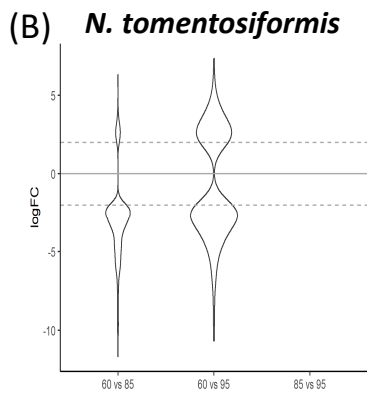

Supplement: Supplementary file 1 [file genes-11-01097-s001.zip › Supplemental Figure S7.pdf]

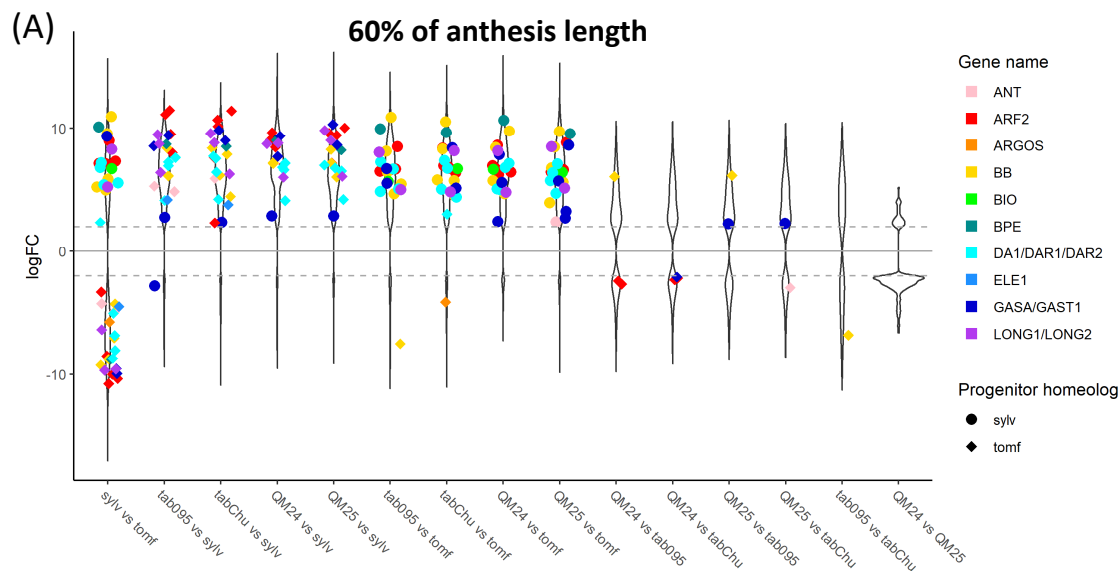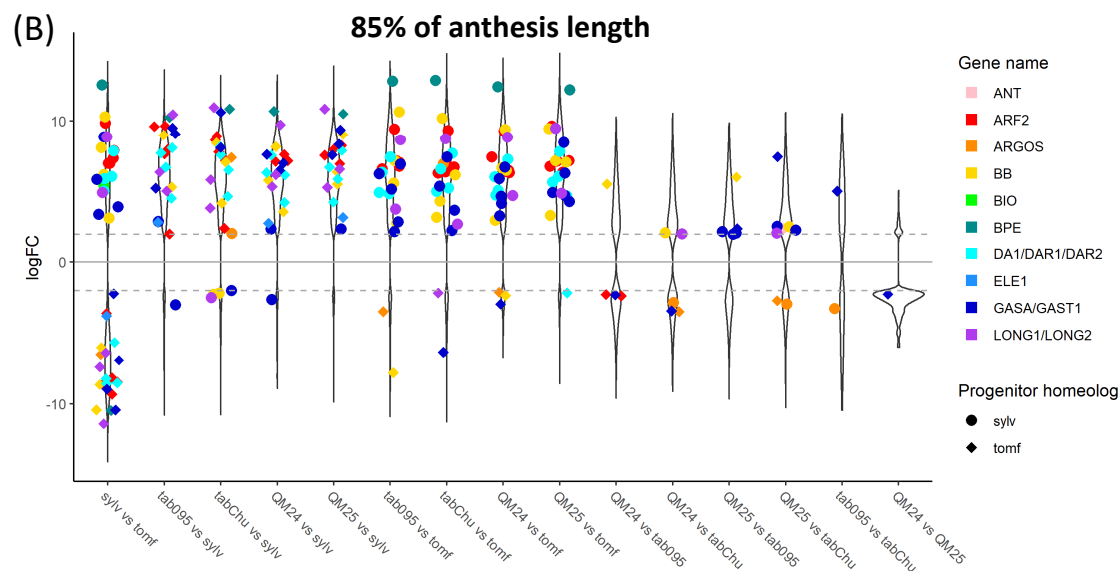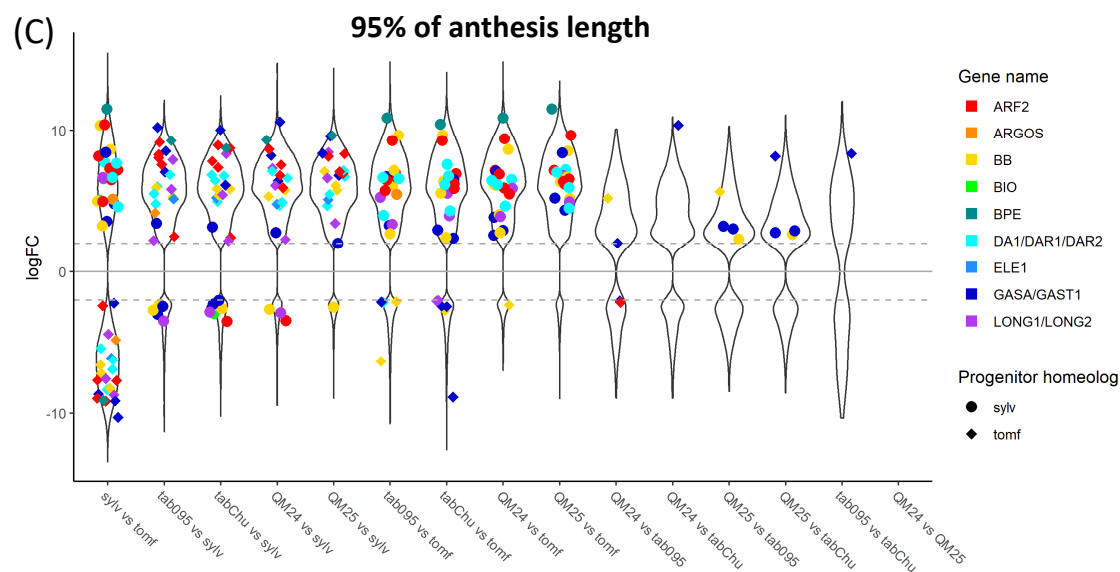

Supplement: Supplementary file 1 [file genes-11-01097-s001.zip › Supplemental Figure S8.pdf]
